# Dosimetric impact of endoscopic titanium clips in photon and proton radiotherapy for esophageal cancer

**DOI:** 10.3389/fonc.2026.1769701

**Published:** 2026-03-09

**Authors:** Ningjing Siah, Xiaohui Wang, Xin Li, Pengyuan Qi, Bingyue Wang, Rui Zhou, Chuanchuan Yu, Jun Han, Xiaorong Dong, Sheng Zhang

**Affiliations:** 1 Cancer Center, Union Hospital, Tongji Medical College, Huazhong University of Science and Technology, Wuhan, China; 2 Institute of Radiation Oncology, Union Hospital, Tongji Medical College, Huazhong University of Science and Technology, Wuhan, China; 3 Hubei Key Laboratory of Precision Radiation Oncology, Wuhan, China; 4 Department of Oncology, Yichang Central People’s Hospital and The First College of Clinical Medical Science, China Three Gorges University Yichang, Yichang, China; 5 Oncology Department, The Second People’s Hospital of Yichang, Three Gorges University, Yichang, China; 6 Department of Medical Statistics, School of Public Health, Sun Yat-sen University, Guangzhou, Guangdong, China

**Keywords:** dose perturbation, esophageal cancer, photon radiotherapy, proton radiotherapy, titanium clips

## Abstract

**Background:**

To quantitatively assess the dosimetric influence of endoscopic titanium clips in photon and proton radiotherapy for esophageal cancer and determine whether clip-induced heterogeneities affect target coverage, esophageal wall dose, or plan robustness.

**Methods:**

This retrospective study included 26 patients with esophageal squamous cell carcinoma who underwent metallic clip placement for radiotherapy localization. Photon IMRT/VMAT and proton pencil beam scanning plans were generated with clip densities represented either by native CT values or overridden to 0 HU. Multiple regions of interest, including the clip, 3-mm and 5-mm expansions and their intersections with the esophageal wall, GTV, and PTV, were evaluated. Dose–volume parameters were compared using paired tests and linear mixed-effects models. Proton plans incorporated robust optimization accounting for ±5 mm setup and ±3.5% range uncertainties.

**Results:**

Clip-induced perturbations were small and localized in both modalities. Photon dose differences were ≤0.3 Gy across evaluated structures, while proton variations were ≤0.1 Gy, confined to the immediate clip vicinity. Target coverage remained stable, with all changes ≤1% of the prescription dose. Proton robustness was preserved under uncertainty scenarios, maintaining ≥95% CTV coverage. Differences between modalities were minimal, and although a few metrics reached statistical significance, the absolute dose changes remained small. Esophageal wall dose increases did not exceed 0.2 Gy (<0.5% of prescription), well below established toxicity thresholds.

**Conclusion:**

Endoscopic titanium clips introduce only minor, clinically insignificant dose perturbations in photon and proton esophageal radiotherapy. Proton sensitivity to local heterogeneities is effectively mitigated by robust optimization. These findings support the dosimetric safety and reliability of titanium clips as localization markers in contemporary treatment planning.

## 1 Introduction

Radiotherapy plays a central role in the multidisciplinary management of esophageal cancer, applied in preoperative, definitive, and palliative settings (1). Despite advances in treatment techniques and imaging guidance, local recurrence and suboptimal tumor control remain frequent. These failures may result from both biological factors, such as tumor hypoxia and accelerated repopulation, and technical issues including inaccurate target delineation (2). Accurate definition of the target volume is essential to ensure adequate tumor coverage, minimize radiation to adjacent organs, and improve clinical outcomes (3).

In clinical practice, localization of the primary site can be challenging when tumors shrink after endoscopic submucosal dissection (ESD) or systemic therapy. Conventional imaging such as CT or MRI often fails to visualize the original lesion, increasing the risk of geographical miss. To overcome this limitation, Riepl et al. first described the use of endoscopic clips for gastrointestinal tumor marking in 2000 (4). Since then, metallic clips have been widely used to mark mucosal lesions in esophageal cancer and facilitate accurate localization during radiotherapy planning (5).

However, due to their high atomic number and density, metallic clips may cause CT artifacts that degrade image quality and potentially alter local dose distribution (6, 7). While previous studies in photon-based radiotherapy reported minor to moderate dosimetric effects (8, 9), their clinical relevance in proton therapy remains unclear. Given the unique depth–dose characteristics and sensitivity to heterogeneity of protons, metallic markers may induce more pronounced dose distortions than photons.

Current evidence regarding titanium clips in esophageal radiotherapy is limited. Investigations in other regions, such as dental and spinal sites, have shown clinically meaningful dose perturbations in proton therapy (10, 11). Moreover, Monte Carlo studies highlight that implant composition (e.g., titanium versus carbon fiber) can substantially affect dose distributions across photon, electron, and proton modalities (12, 13). Yet, the specific impact of endoscopically placed titanium clips in the esophagus has not been systematically evaluated.

This study aims to quantitatively compare the dosimetric influence of titanium clips in photon and proton treatment plans for esophageal cancer, providing quantitative evidence to guide dose calculation and plan evaluation in the presence of endoscopic clips.

## 2 Materials and methods

### 2.1 Patient selection

This retrospective study included patients with histologically confirmed esophageal squamous cell carcinoma (ESCC) who underwent metallic clip placement for radiotherapy target localization at our institution between November 2021 and March 2024. Eligible patients were required to have clips placed during endoscopy and to have completed CT simulation for radiotherapy planning. Patients were excluded if clip placement was not performed within the target volume, if imaging data were incomplete, or if radiotherapy planning data were unavailable for dosimetric analysis. A total of 26 patients met these criteria and were included in the study.

This study was conducted in accordance with the Declaration of Helsinki and was approved by the institutional review board (IRB) of Union Hospital, Huazhong University of Science and Technology (Approval No.20240832). Given the retrospective design and the use of anonymized clinical data, the requirement for individual informed consent was waived by the IRB.

### 2.2 Endoscopic metallic clips

Endoscopic metallic clips were used to mark the tumor bed and guide radiotherapy target delineation. Placement followed standardized procedures (1): clips were anchored on adjacent normal mucosa to reduce dislodgement risk (2); clips were oriented perpendicular to the esophageal wall to improve visibility and reproducibility (3); CT simulation was performed within 4 hours after endoscopy to confirm clip position; and (4) the superior and inferior clip edges defined the corresponding target margins.

Rotatable endoscopic tissue clips were deployed using a compatible rotatable clip system (SureClip™ series, ROcc-D-26-195-C, Nanjing Micro-Tech Medical Company, Nanjing, China). The implanted clips were titanium-based endoscopic clips designed for endoscopic use and long term biocompatibility. As detailed material specifications of the implanted clips are not publicly available, a material density of 4.5 g/cm³ was assumed based on standard reference values for titanium materials, which is consistent with the reported density of commercially pure titanium (~4.51 g/cm³). Due to their radiopaque property, clips were easily visualized on CT scans. In treatment planning, clips were represented with their native CT density values in the original plans and overridden to 0 HU in modified plans to assess their dosimetric influence. This approach minimized the dependence of the analysis on the exact material density of the clips.

### 2.3 Treatment planning

CT simulation was performed with patients in the supine position using a wide-bore CT scanner (Brilliance CT Big Bore, Philips Healthcare, Netherlands). A customized vacuum cushion and thermoplastic mask were applied for immobilization. CT images were reconstructed with 3-mm slice thickness for planning. The gross tumor volume (GTV), clinical target volume (CTV) and planning target volume (PTV) were delineated according to the Radiation Therapy Oncology Group guidelines, and organs at risk (OARs) were first contoured automatically and then refined manually. All contours were verified by experienced radiation oncologists.

**Figure 1:**
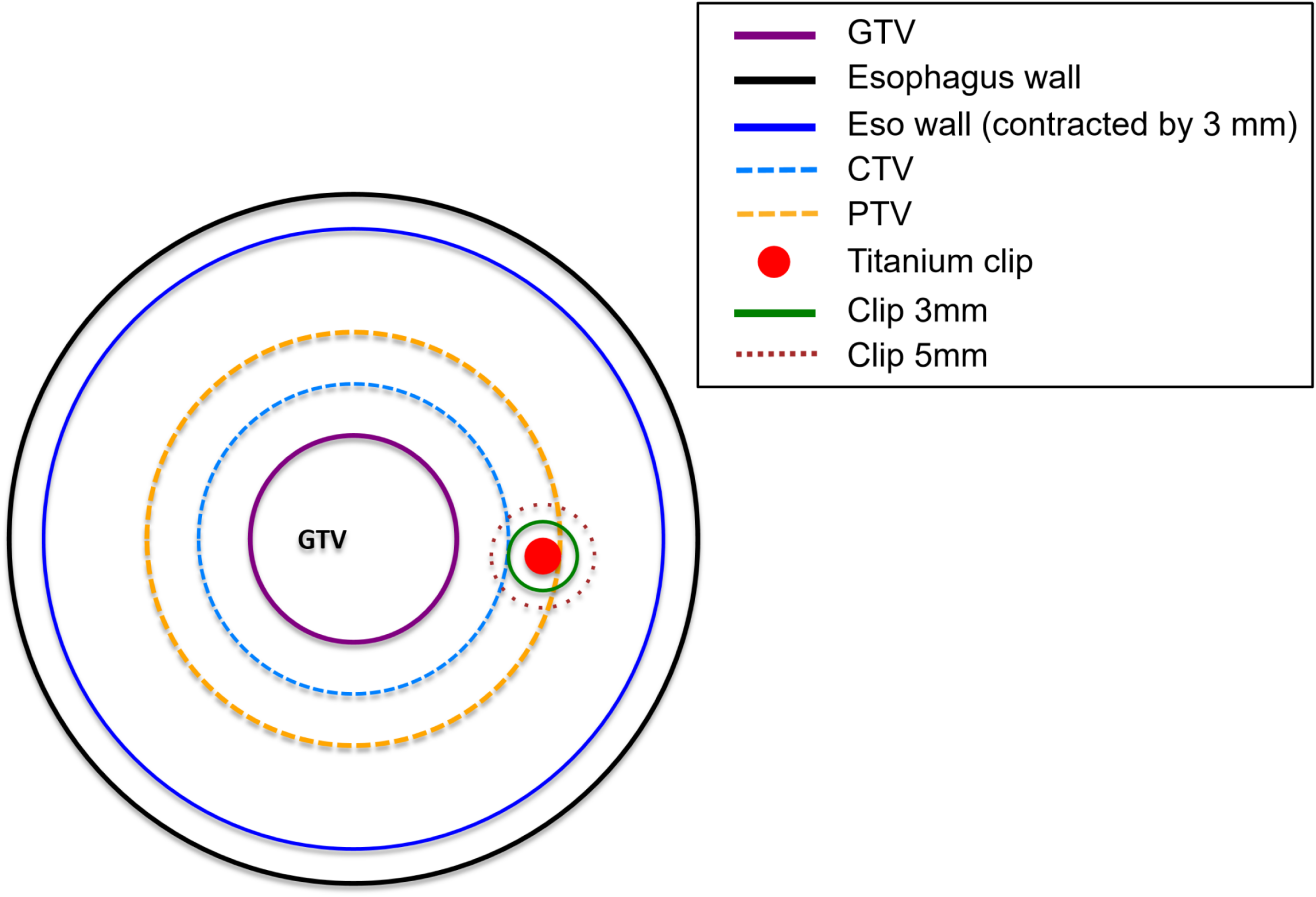
Schematic illustration of the geometric arrangement of the regions of interest (ROIs). The gross tumor volume (GTV), clinical target volume (CTV), and planning target volume (PTV) are shown together with the esophageal wall and the esophageal wall contracted inward by 3 mm. The titanium clip and its 3-mm and 5-mm isotropic expansions (Clip 3mm and Clip 5mm) are also indicated. Detailed definitions of all ROIs are provided in **Supplementary Table 1**.

To evaluate clip-induced dose perturbations, multiple regions of interest (ROIs) were generated. The esophageal wall (Eso wall) was defined by contracting the external contour of the esophagus by 3 mm, and its overlap with the PTV was designated as Eso wall in PTV. Metallic clips (Titanium clip) and their 3 mm and 5 mm isotropic expansions were labeled as Clip 3 mm and Clip 5 mm, respectively. Their intersections with the esophageal wall, GTV, and PTV formed additional analysis structures, as summarized in **Supplementary Table 1** and illustrated schematically in **Figure 1**.

Photon and proton treatment plans were initially generated with the clips assigned their actual high density CT values (native plans). Photon plans were optimized in Eclipse treatment planning system (v15.1, Varian Medical Systems, Palo Alto, CA, USA) using 6MV beams employing intensity modulated radiotherapy (IMRT) or volumetric-modulated arc therapy (VMAT), ensuring ≥95% PTV coverage. Geometric uncertainties, including patient setup errors, were accounted through the CTV-to-PTV margin expansion, consistent with standard photon radiotherapy practice. Dose calculation was performed by the Acuros XB algorithm. Proton plans were developed in Eclipse (v16.1, Varian Medical Systems) using pencil beam scanning (PBS) with a 3 to 4 field arrangement to achieve ≥95% prescribed dose coverage. Dose calculation was performed using the Proton Convolution Superposition (PCS) algorithm. Robust optimization was performed on the CTV, accounting for ±5 mm setup and ±3.5% range uncertainty. For cross-modality dose comparison, a pseudo-PTV (CTV + 5 mm) was generated for proton plans, mimicking the photon PTV.

For density-modified plans, clip CT values were overridden to 0 HU, with all beam parameters unchanged. Dose distributions were recalculated using the respective photon and proton algorithms. All plans were created by experienced physicists and reviewed by senior radiation oncologists.

### 2.4 Dosimetric analysis

**Figure 2:**
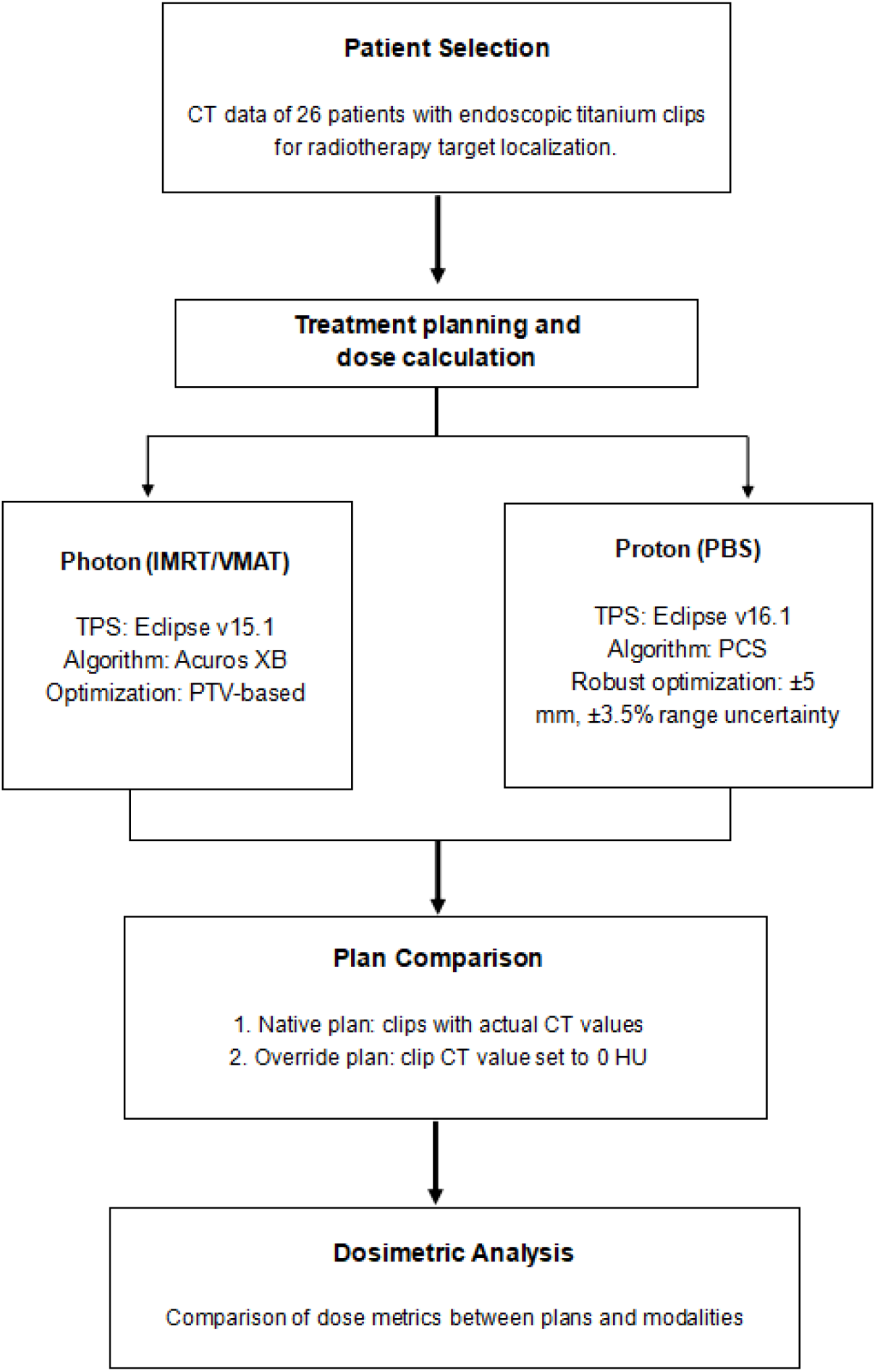
Workflow of the study design and dosimetric analysis. Photon (IMRT/VMAT) and proton (PBS) treatment plans were generated for 26 patients with endoscopic titanium clips for radiotherapy target localization. Native plans incorporated the actual CT density values of the clips, whereas override plans assigned the clip CT values to 0 HU. Dose metrics were subsequently compared between native and override plans, as well as between photon and proton modalities.

Dosimetric parameters were evaluated for both target volumes and clip associated regions. For the PTV and GTV, D95 and Dmax were recorded under native and override clip density conditions. To assess dose deposition within the clip, Dmean, Dmax, and D0.1cc were calculated. Surrounding tissue exposure was analyzed by generating 3-mm and 5-mm isotropic expansions of the clip, with the same metrics extracted. For both expansions, the overlapping portions with the esophageal wall were delineated (Clip 3mm in esowall and Clip 5mm in esowall) and evaluated separately. Differences and absolute differences were calculated between native and override states and between photon and proton modalities. The overall workflow of treatment planning, plan recalculation, and dosimetric analysis is summarized in **Figure 2**. For patients with a single clip, all clip-related ROIs (including the clip itself, 3-mm and 5-mm isotropic expansions, and their intersections with the esophageal wall, GTV, and PTV) were generated from that clip alone, and all dose metrics were analyzed at the patient level.

### 2.5 Statistical analysis

Statistical analyses were performed using R software (version 4.4.1). Dosimetric parameters were reported as mean ± standard deviation (SD). Paired t-tests were used for direct comparisons between conditions, while linear mixed-effects models were applied to evaluate the main effects of modality (photon vs proton) and clip modeling (native vs override), with patient specified as a random effect. Both dose differences (Δ) and relative percentage difference (Δ%) were analyzed and visualized using box plots and waterfall plots generated with the ggplot2 package. All tests were two-sided, and p-value < 0.05 was considered statistically significant.

## 3 Results

### 3.1 Patient and treatment overview

A total of 26 patients with ESCC were analyzed. The median age was 64 years (range, 46–75), and most were male (84.6%) with good performance status (ECOG 0–1, 92.3%). Tumors were mainly located in the lower thoracic esophagus (65.4%). According to the AJCC 8th edition, 34.6% were stage IB, 23.2% stage IIA, 19.2% stage IIB, 19.2% stage IIIA, and 3.8% stage IIIB. For photon-based radiotherapy, 15 patients (57.7%) received IMRT and 11 (42.3%) received VMAT, while proton therapy plans were generated using the Varian ProBeam system. Prescribed doses ranged from 40 to 66 Gy in 20–33 fractions, with 42.3% received conventional fractionation, 38.5% moderate hypofractionation, and 19.2% hypofractionated regimens. Metallic clips were placed at the superior and inferior tumor margins for target localization, while three patients had only one clip placed due to severe stenosis and were included in all analyses. Detailed clinical and treatment characteristics are presented in **Table 1**.

**Table 1:** Patient demographics and disease characteristics.

Characteristic	Total (n = 26)	Percentage (%)
Age, years (median, range)	64 (46–75)	
Sex		
Male	22	84.6
Female	4	15.4
ECOG Performance Status		
0–1	24	92.3
2	2	7.7
Tumor Location		
Upper thoracic esophagus	3	11.5
Middle thoracic esophagus	6	23.1
Lower thoracic esophagus	17	65.4
Histology		
Squamous cell carcinoma	26	100
T stage		
T1	10	38.5
T2	6	23.1
T3	7	26.9
T4	3	11.5
N stage		
N0	10	38.5
N1	9	34.6
N2	6	23.1
N3	1	3.8
M stage		
M0	26	100
Overall Stage (AJCC 8th)		
Stage IB	9	34.6
Stage IIA	6	23.2
Stage IIB	5	19.2
Stage IIIA	5	19.2
Stage IIIB	1	3.8
Treatment technique for photon		
IMRT	15	57.7
VMAT	11	42.3
Radiotherapy Fractionation		
Conventional fractionation (60–66 Gy in 30–33 F)	11	42.3
Moderate hypofractionation (52–58 Gy in 26–29 F)	10	38.5
Hypofractionated regimen (40 Gy/20 F)	5	19.2

ECOG, Eastern Cooperative Oncology Group; IMRT, intensity-modulated radiation therapy; VMAT, volumetric modulated arc therapy; Gy, gray; F, fraction.

### 3.2 Clip-induced dose perturbations

**Figure 3:**
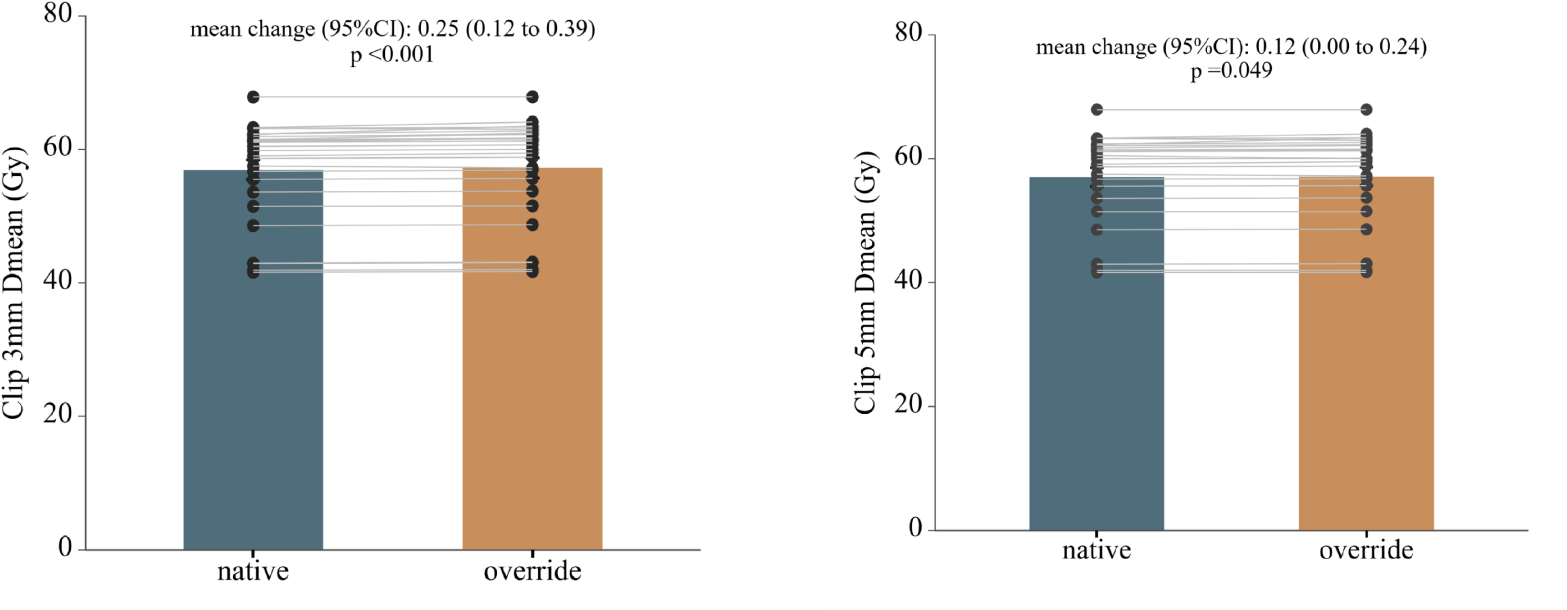
Photon plans. Paired comparison of mean dose (Dmean) before and after CT-number override in photon treatment plans for regions surrounding metallic clips. Panels show Clip 5 mm and Clip 3 mm regions, respectively. Bars represent mean ± standard deviation, and thin gray lines indicate paired patient level data. Mean changes with 95% confidence intervals and corresponding p-values were derived from paired t-tests.

**Figure 4:**
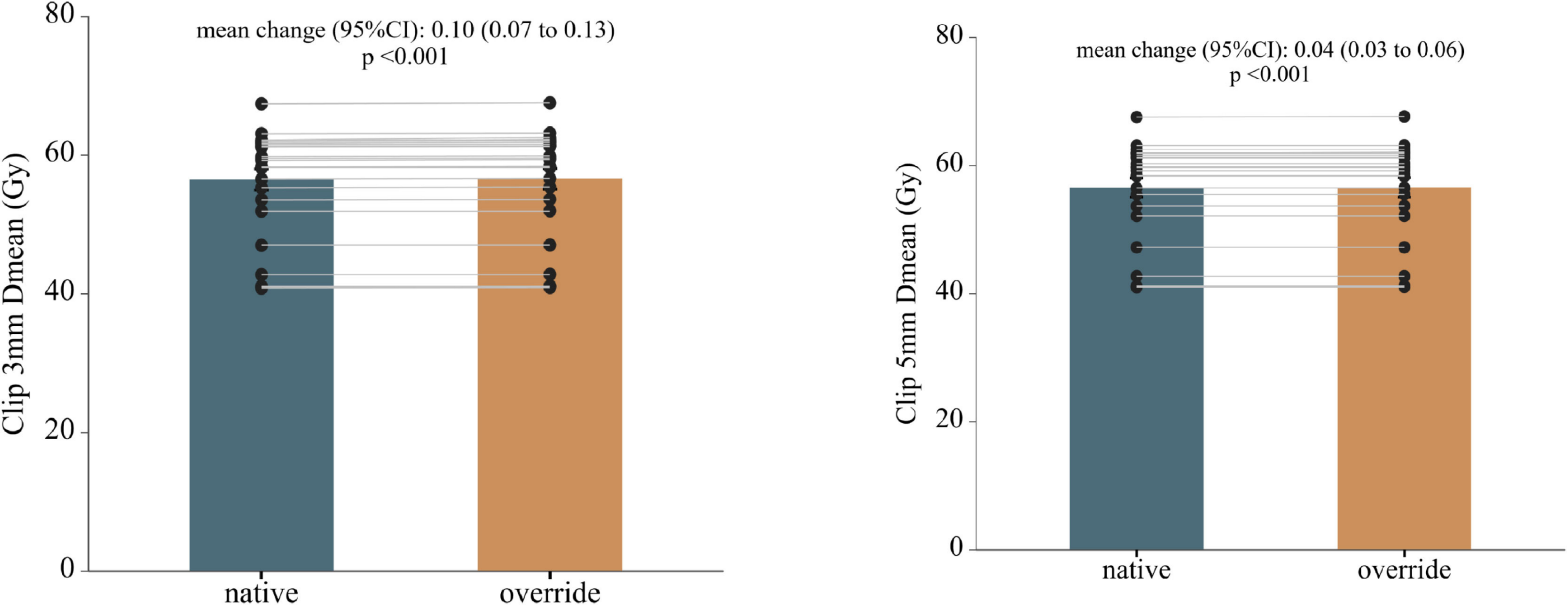
Proton plans. Paired comparison of mean dose (Dmean) before and after CT-number override in proton treatment plans for regions surrounding metallic clips. The panel layout, color scheme, and statistical annotations are identical to those in **Figure 3**, enabling direct visual comparison between photon and proton modalities.

**Figure 5:**
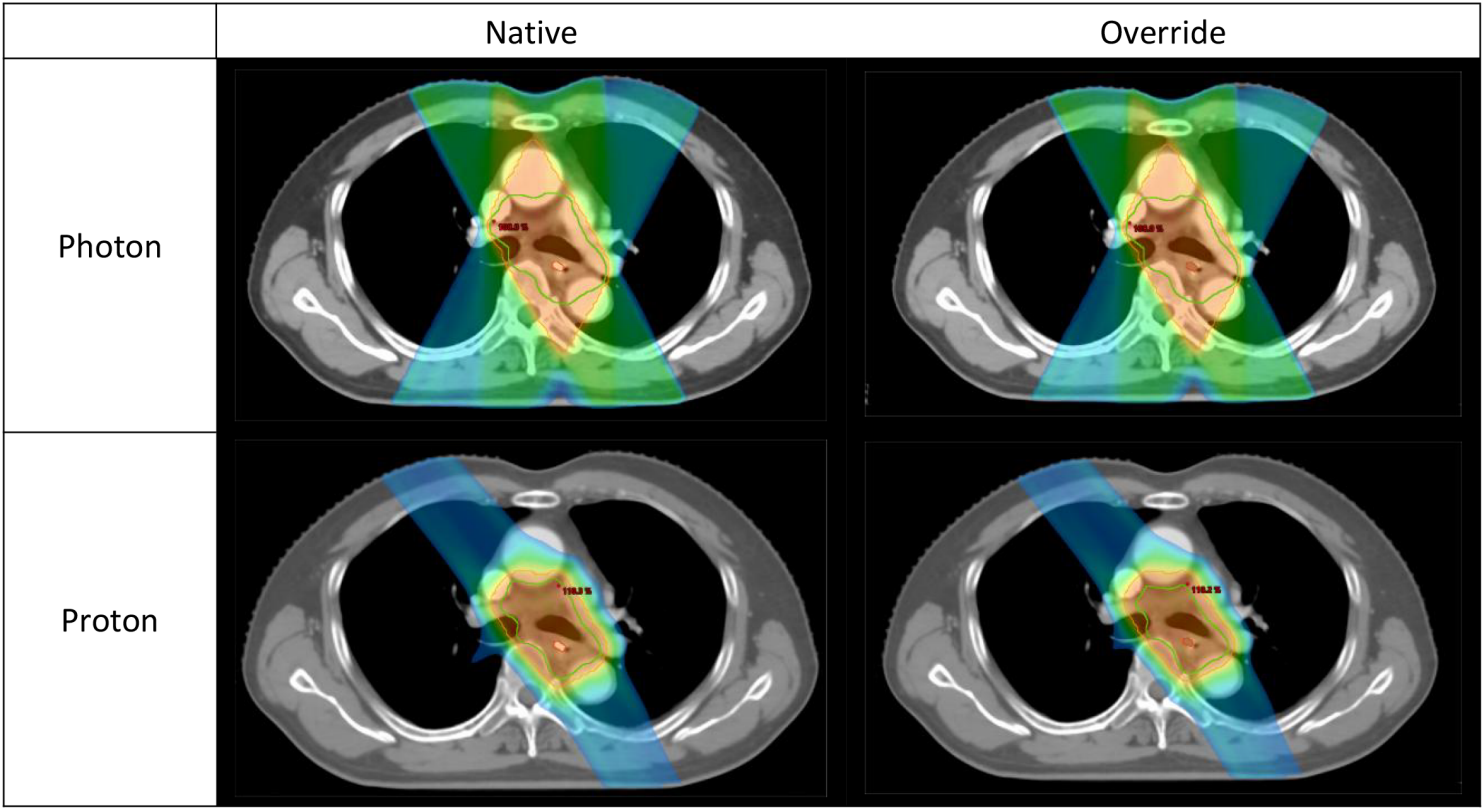
Representative dose distributions in photon and proton radiotherapy. Representative axial dose distributions for the same patient comparing photon and proton radiotherapy plans calculated on native CT images and with titanium clip density override. The upper row shows photon plans and the lower row shows proton plans; the left column represents native plans and the right column represents override plans. The orange region denotes the 95% isodose, the green contour indicates the PTV in photon plans and the CTV in proton plans, and the red contour marks the endoscopic titanium clip. The dose distributions visually support the quantitative results in **Tables 2** and **3**.

Following CT-number override, dose recalculation revealed localized but statistically significant dose increases in both photon and proton plans (**Table 2**; **Supplementary Table 2**). In absolute terms, the magnitude of clip-induced dose perturbations was small in both modalities. Within photon plans, mean dose differences between native and override calculations were ≤ 0.3 Gy across all clip-related structures. Statistically significant increases were observed in Clip 3 mm Dmean (Δ = 0.25 Gy, p < 0.001) and Clip 5 mm Dmean (Δ = 0.12 Gy, p = 0.049). Dose elevations were also detected at the clip–esophageal wall interface, with increased Dmean values for both Clip 3 mm and Clip 5 mm in EsoWall regions (p < 0.05), whereas high-dose subvolumes (D0.1cc) remained unchanged (p > 0.5). In proton plans, consistent but smaller absolute dose elevations were observed surrounding the clips, with Clip 3 mm Dmean increased by 0.10 Gy (p < 0.001) and Clip 5 mm Dmean by 0.04 Gy (p < 0.001). As illustrated in **Figures 3** and **4**, these differences were statistically significant but of small absolute magnitude, confirming that dose perturbations were confined to the immediate clip vicinity. Representative axial dose distributions illustrating the spatial characteristics of clip-induced dose perturbations in photon and proton plans are shown in **Figure 5**.

**Table 2:** Paired comparison of target and clip-related dose parameters between native and override plans for photon and proton therapy.

Structure/Parameter	Photon plans				Proton plans			
	Native	Override	Δ (95% CI)	p	Native	Override	Δ (95% CI)	p
Target Volume								
PTV D95 (%)	101.28 ± 1.42	101.39 ± 1.43	0.11 (-0.05 to 0.26)	0.160	90.86 ± 3.95	90.94 ± 3.96	0.08 (0.04 to 0.12)	< 0.001*
PTV with Clip D95 (Gy)	55.89 ± 7.34	56.23 ± 7.47	0.35 (0.15 to 0.55)	0.001*	55.12 ± 7.50	55.17 ± 7.48	0.05 (-0.04 to 0.14)	0.247
GTV D95 (%)	102.35 ± 1.67	102.51 ± 1.67	0.16 (0.01 to 0.31)	0.038*	101.06 ± 0.64	101.10 ± 0.64	0.03 (0.00 to 0.06)	0.029*
GTV with Clip D95 (Gy)	55.88 ± 7.35	56.31 ± 7.54	0.44 (0.20 to 0.68)	< 0.001*	55.32 ± 7.59	55.40 ± 7.60	0.08 (0.02 to 0.13)	0.007*
Clip-related regions								
Clip 3mm Dmean (Gy)	56.97 ± 7.51	57.22 ± 7.60	0.25 (0.12 to 0.39)	< 0.001*	56.51 ± 7.67	56.60 ± 7.70	0.10 (0.07 to 0.13)	< 0.001*
Clip 3mm D0.1cc (Gy)	58.30 ± 7.81	58.38 ± 7.83	0.09 (-0.05 to 0.22)	0.221	58.18 ± 7.87	58.26 ± 7.92	0.09 (-0.02 to 0.19)	0.113
Clip 3mm in EsoWall Dmean (Gy)	56.91 ± 7.53	57.22 ± 7.62	0.31 (0.14 to 0.48)	< 0.001*	56.52 ± 7.62	56.66 ± 7.66	0.14 (0.09 to 0.20)	< 0.001*
Clip 5mm Dmean (Gy)	57.04 ± 7.53	57.16 ± 7.58	0.12 (0.00 to 0.24)	0.049*	56.55 ± 7.65	56.59 ± 7.66	0.04 (0.03 to 0.06)	< 0.001*
Clip 5mm D0.1cc (Gy)	58.52 ± 7.88	58.42 ± 7.82	-0.10 (-0.46 to 0.26)	0.571	58.64 ± 7.91	58.71 ± 7.99	0.07 (-0.06 to 0.20)	0.268
Clip 5mm in EsoWall Dmean (Gy)	56.98 ± 7.42	57.17 ± 7.59	0.19 (0.01 to 0.38)	0.041*	56.58 ± 7.67	56.63 ± 7.69	0.06 (0.03 to 0.08)	< 0.001*

Δ= difference between override and native plans (override – native). Data are presented as mean ± SD. p-values were obtained using paired t-tests.

Gy, gray; D95, dose to 95% of the volume; Dmean, mean dose; D0.1cc, minimum dose delivered to the hottest 0.1 cc; EsoWall, esophageal wall.

* indicate statistical significance (p < 0.05).

### 3.3 Effects on target and esophageal wall dosimetry

Target coverage metrics remained stable after CT-number override (**Table 2**). For photon plans, PTV D95 (101.3 ± 1.4% vs 101.4 ± 1.4%) and GTV D95 (102.4 ± 1.7% vs 102.5 ± 1.7%) showed minimal variation (Δ ≤ 0.2%, p > 0.05). However, PTV with Clip D95 and GTV with Clip D95 increased slightly but significantly (Δ = 0.35 Gy, p = 0.001; Δ = 0.44 Gy, p < 0.001, respectively). Similarly, in proton plans, PTV D95 rose by 0.08% (p < 0.001) and GTV D95 by 0.03% (p = 0.029), while GTV with Clip D95 showed a minor yet significant increase (Δ = 0.08 Gy, p = 0.007). Although several parameters showed statistically significant differences after CT-number override, all changes were within 1% of the prescribed dose, and no clinically relevant deterioration in dose uniformity or target coverage was observed, confirming that clip-induced perturbations did not compromise plan quality.

### 3.4 Photon–proton differences

When comparing dose differences (Δ = override – native) between photon and proton plans, the overall magnitude of clip-induced perturbations was small across most parameters (**Table 3**). Only a few metrics showed statistically significant inter-technique differences, the most evident being Clip 3 mm Dmean, where proton plans exhibited greater local dose elevation than photon plans (p = 0.039). PTV with Clip D95 and GTV with Clip D95 also showed small but significant inter-modality differences (p < 0.05), both indicating slightly higher perturbations in proton therapy. In contrast, most esophageal wall related parameters showed no meaningful inter-modality variation, except for EsoWall in PTV Dmean, which demonstrated a borderline yet statistically significant difference (p = 0.050), with slightly higher values in photon plans. Overall, photon dose distributions remained stable, whereas proton plans showed slightly larger localized perturbations within the 3-mm region surrounding the clips, without any significant discrepancies in esophageal wall dose or overall target coverage. Photon–proton comparisons are summarized in **Table 3**, with detailed structure-specific results provided in the **Supplementary Material**.

**Table 3:** Comparison of clip induced dose perturbations between photon and proton plans after CT-number override.

Structure/Parameters	Photon		Proton		Difference (95% CI) (Gy)	Difference (95% CI) (%)	p (Gy)	p (%)
	Δ (Gy)	Δ%	Δ (Gy)	Δ%				
Target Volume								
PTV D95 (%)	0.11 ± 0.38	0.11 ± 0.38	0.08 ± 0.10	0.08 ± 0.11	-0.03 (-0.18 to 0.12)	-0.02 (-0.17 to 0.13)	0.677	0.760
PTV with Clip D95 (Gy)	0.35 ± 0.49	0.60 ± 0.82	0.05 ± 0.21	0.10 ± 0.36	-0.30 (-0.54 to -0.05)	-0.50 (-0.91 to -0.10)	0.019*	0.017*
GTV D95 (%)	0.16 ± 0.38	0.16 ± 0.37	0.03 ± 0.07	0.03 ± 0.07	-0.13 (-0.28 to 0.02)	-0.13 (-0.28 to 0.02)	0.092	0.091
GTV with Clip D95 (Gy)	0.44 ± 0.59	0.75 ± 1.00	0.08 ± 0.13	0.14 ± 0.23	-0.36 (-0.61 to -0.11)	-0.61 (-1.03 to -0.19)	0.006*	0.006*
Metallic Clip Region								
Clip 5mm D0.1cc (Gy)	-0.10 ± 0.90	-0.15 ± 1.40	0.07 ± 0.31	0.10 ± 0.48	0.17 (-0.21 to 0.55)	0.25 (-0.34 to 0.84)	0.365	0.393
Clip 5mm in EsoWall Dmean (Gy)	0.19 ± 0.46	0.31 ± 0.75	0.06 ± 0.05	0.10 ± 0.09	-0.14 (-0.32 to 0.04)	-0.21 (-0.51 to 0.08)	0.126	0.153
Clip 5mm in EsoWall D0.1cc (Gy)	-0.06 ± 0.83	-0.07 ± 1.30	0.09 ± 0.46	0.16 ± 0.78	0.15 (-0.23 to 0.53)	0.23 (-0.37 to 0.84)	0.426	0.439
Clip 3mm Dmean (Gy)	0.25 ± 0.33	0.43 ± 0.52	0.10 ± 0.07	0.17 ± 0.12	-0.15 (-0.30 to -0.01)	-0.26 (-0.49 to -0.02)	0.039*	0.032*
Clip 3mm D0.1cc (Gy)	0.08 ± 0.34	0.14 ± 0.57	0.09 ± 0.27	0.14 ± 0.42	0.00 (-0.18 to 0.18)	-0.01 (-0.30 to 0.29)	0.990	0.966
Clip 3mm in EsoWall Dmean (Gy)	0.31 ± 0.42	0.54 ± 0.68	0.14 ± 0.14	0.25 ± 0.22	-0.16 (-0.35 to 0.02)	-0.28 (-0.58 to 0.01)	0.075	0.061
Clip 3mm in EsoWall D0.1cc (Gy)	0.20 ± 0.37	0.34 ± 0.61	0.07 ± 0.27	0.13 ± 0.43	-0.13 (-0.30 to 0.05)	-0.22 (-0.50 to 0.07)	0.156	0.137
Esophagus Region								
EsoWall V60 (%)	0.28 ± 0.59	0.38 ± 22.04	0.01 ± 0.15	-2.01 ± 8.15	-0.27 (-0.54 to 0.00)	-2.38 (-15.14 to 10.38)	0.052	0.698
EsoWall in PTV Dmean (Gy)	0.10 ± 0.27	0.17 ± 0.44	-0.10 ± 0.56	-0.16 ± 0.90	-0.20 (-0.40 to 0.00)	-0.33 (-0.65 to -0.00)	0.050*	0.049*
EsoWall in PTV D0.1cc (Gy)	0.04 ± 0.34	0.06 ± 0.54	-0.07 ± 0.36	-0.12 ± 0.57	-0.11 (-0.25 to 0.03)	-0.18 (-0.39 to 0.03)	0.108	0.090

Δ represents the mean paired difference between override and native plans (override – native); Δ% the relative percentage difference. Difference (95% CI) represents the mean difference between photon and proton Δ values.

Separate p values are reported for absolute (Gy) and relative (%) differences.

* indicate statistical significance (p < 0.05).

### 3.5 Interaction and robustness analyses

Linear mixed effects modeling revealed no significant interaction between radiotherapy modality (photon vs proton) and plan type (native vs override) for most dose parameters (p > 0.05; **Supplementary Table 4**). The only notable interaction was observed for Clip Dmean (β = –0.8, 95% CI –1.48 to –0.12, p = 0.024), indicating that dose changes around the clips differed slightly between photon and proton plans. For all other target and esophageal wall related metrics, interaction terms were non-significant, suggesting that the relative impact of CT-number override was consistent across both modalities.

Sensitivity analyses confirmed overall stability of dose distributions against CT-number variations induced by metallic clips. Across both modalities, no clinically relevant deterioration in target coverage or dose conformity was observed after density override. In photon plans, dose–volume indices for PTV and GTV remained within ±0.3 Gy, and the influence of clips on surrounding esophageal structures was negligible. For proton therapy, although localized perturbations near the clips were statistically significant, their absolute magnitudes were small (Δ ≤ 0.1 Gy) and did not compromise robust optimization outcomes. All proton plans maintained ≥95% CTV coverage under ±5 mm setup and ±3.5% range uncertainty conditions, confirming robust dose delivery even in the presence of titanium markers. Overall, these findings support the dosimetric safety and robustness of both photon and proton treatment plans, indicating that titanium clips do not introduce clinically meaningful risks to target coverage or esophageal wall sparing in current planning frameworks.

## 4 Discussion

This study systematically evaluated the dosimetric influence of endoscopic titanium clips in photon and proton radiotherapy for esophageal cancer. Clip-induced perturbations were localized, small in magnitude, and clinically negligible. Although several parameters reached statistical significance, all absolute dose differences remained within 1% of the prescribed dose (≤ 0.3 Gy for photon therapy and ≤ 0.1 Gy for proton therapy). From a clinical planning standpoint, these variations are well within commonly accepted target coverage objectives, where CTV D95 is typically required to be ≥95% of the prescription dose, confirming that titanium clips do not compromise clinically acceptable target coverage. These results extend previous phantom-based findings to a patient specific setting and provide new patient-based quantitative evidence in esophageal radiotherapy.

In photon therapy, the minimal perturbations observed align with earlier reports showing <1% dose deviation near titanium implants in megavoltage beams (14, 15). The consistency of our data (Δ ≤ 0.3 Gy) reflects the accuracy of advanced dose calculation algorithms such as Acuros XB, which effectively model photon–metal interactions (16, 17). Consequently, photon dose distributions remained stable even in clip adjacent regions, supporting the robustness of IMRT and VMAT planning. Importantly, unlike studies focusing on large spinal or dental implants, endoscopic titanium clips represent small volume metallic markers with limited thickness and effective beam path length. Their small size, thin geometry, and intraluminal or intramural location explain why the resulting dose perturbations are substantially smaller and more localized than those reported for bulky orthopedic or dental implants (18, 19).

In proton therapy, metallic heterogeneities may theoretically exert a greater influence due to the finite range and sharp distal fall-off of proton beams. However, most prior studies addressing metal induced perturbations in proton therapy have focused on large, high density implants that intersect multiple beam paths or located near high gradient distal regions. In contrast, endoscopic clips used for esophageal cancer are typically small (<1 cm³), thin, and embedded within soft tissue. This fundamental difference in size and geometry explains why dose perturbations in the present study were confined to the immediate clip vicinity and remained far below levels reported for large orthopedic or dental devices (20, 21). From a proton range perspective, the clip-induced changes in water-equivalent depth (WED) were very small (<0.5 mm), reflecting the limited physical size and localized nature of the clips. Such minimal WED variations are insufficient to induce a meaningful proton range shift along the beam path and therefore remain well within the robustness envelope of clinically optimized proton plans.

In routine clinical proton planning for esophageal cancer, endoscopic clips are unlikely to systematically coincide with the distal edge of proton beams. All proton plans maintained ≥95% CTV coverage under ±5 mm setup and ±3.5% range uncertainties, demonstrating that robust optimization effectively mitigates the dosimetric impact of local density variations. Similar conclusions have been reported in prior studies showing that robust optimization reduces heterogeneity driven uncertainties in proton dose calculations (22, 23). The borderline difference observed in EsoWall PTV Dmean between photon and proton plans (p = 0.050), with slightly higher values in photons, is more likely due to inherent differences in dose calculation algorithms rather than a clinically meaningful clip-related effect.

The significant interaction observed for Clip Dmean in the interaction analysis likely reflects fundamental differences in particle matter interactions between photon and proton beams. In photon therapy, dose perturbations near small metallic objects are primarily governed by Compton scattering and secondary electron transport, leading to relatively smooth and spatially averaged dose changes within small volumes. In contrast, proton dose deposition is influenced by multiple Coulomb scattering and local variations in stopping power, which can alter proton energy loss and lateral scattering in the immediate vicinity of high density materials. These mechanisms may result in a different response pattern of mean dose within the clip adjacent region following density override, even when the absolute magnitude of the perturbation remains small (24, 25). Importantly, this interaction effect was confined to Clip Dmean and did not translate into clinically relevant changes in target coverage or high dose subvolumes.

Taken together, these results indicate that despite differing physical interaction mechanisms, the dosimetric impact of small endoscopic titanium clips remains minimal in both photon and proton radiotherapy. Clip migration following endoscopic placement represents a potential clinical consideration. However, published studies on endoscopic clip-based localization in esophageal cancer suggest that metallic clips generally remain sufficiently stable for clinical use within the esophageal wall or lumen (26). Given the highly localized and sub-millimeter scale of the dose perturbations observed in the present study, minor clip displacement is unlikely to result in clinically meaningful dosimetric changes, particularly in the presence of setup margins and robust optimization strategies routinely applied in current clinical practice.

From a clinical perspective, even in the most affected regions, dose increases were minimal and did not exceed 0.2 Gy (<0.5% of the prescription), remaining well below established thresholds for esophageal toxicity reported in normal tissue tolerance data (27). Clinically relevant radiation esophagitis has been associated with high dose esophageal dose volume parameters such as V50 and V60, as summarized in QUANTEC analyses and further confirmed in more recent IMRT-based clinical studies (28, 29). In contrast, the clip-induced dose increases observed in the present study were highly localized and of insufficient magnitude to meaningfully influence esophageal toxicity risk. Accordingly, endoscopic titanium clips, which serve as radiopaque fiducial markers, can be safely incorporated into image-guided radiotherapy (IGRT) workflows for target localization and treatment verification. Moreover, the combined photon–proton analysis showed comparable dosimetric behavior with and without CT number override, indicating that routine CT number override of endoscopic titanium clips is not strictly necessary for standard photon or proton esophageal radiotherapy planning when appropriate margins and robust optimization are applied, although override may still be considered in selected proton cases where clips are located near critical beam range regions.

The strengths of this study include its patient-based design, parallel evaluation of photon and proton modalities, and the use of a spatially resolved region of interest framework that enabled detailed assessment of clip-adjacent and esophageal wall dose effects. However, several limitations should be acknowledged. Respiratory motion and organ deformation were not modeled using 4D-CT or ITV-based motion management strategies; accordingly, the findings should be interpreted within the defined dosimetric sensitivity-analysis framework. Dose calculations were based on commercial treatment planning system algorithms (Acuros XB for photons and pencil beam algorithm for protons), which, although widely validated, may not fully capture peak dose perturbations at metal–tissue interfaces, particularly at the sub-millimeter scale. This limitation could lead to a slight underestimation of highly localized dose perturbations immediately adjacent to the titanium clips, especially in proton therapy. Future studies incorporating Monte Carlo simulations or experimental dosimetric measurements would provide more definitive validation of these findings.

In addition, the clip removal scenario was simulated by overriding the clip CT number to 0 HU, representing a simplified approximation rather than a true anatomical “no-clip” condition. In reality, the space previously occupied by the clip would be replaced by soft tissue (~40 HU). However, given the small clip volume and the minimal absolute dose differences observed in this study, the dosimetric impact of using 0 HU instead of soft tissue density is expected to be negligible, particularly with respect to proton range and target coverage.

Finally, the patient cohort demonstrated relatively consistent clip numbers, locations, and spatial relationships to beam paths. Caution is therefore warranted when extrapolating these results to clinical scenarios involving a larger number of clips, clips positioned within high gradient regions such as the distal edge of a proton beam, or clips composed of higher density materials (e.g., stainless steel). Further investigations are needed to explore these less common but potentially more challenging situations.

In summary, endoscopic titanium clips produce only small and localized dose perturbations in both photon and proton esophageal radiotherapy. Although proton dose deposition is governed by different physical interaction mechanisms, robust optimization effectively compensates for local heterogeneities, resulting in only minimal and clinically negligible dose perturbations. Overall, titanium clips can be considered dosimetrically safe and reliable markers for contemporary photon and proton treatment planning.

## Data Availability

The raw data supporting the conclusions of this article will be made available by the authors, without undue reservation.
