# Supplementary material for: Dosimetric impact of endoscopic titanium clips in photon and proton radiotherapy for esophageal cancer
Source: Front Oncol. 2026 Mar 9;16:1769701. doi: 10.3389/fonc.2026.1769701 (PMC13006252; doi:10.3389/fonc.2026.1769701)
Supplement: Supplementary file 1 [file DataSheet1.pdf]

Supplementary Table S1. Definition of the ROIs used for dosimetric evaluation

Structure Name	Definition
Eso wall (contracted by 3 mm)	Contracted inward by 3 mm from esophagus
Esowall in PTV	Portion of the esophageal wall located within the PTV
Titanium clip	Metallic clip marker
Clip 3mm	A 3-mm isotropic expansion around the metallic clip
Clip 3mm in esowall	Intersection between the 3-mm clip expansion and the esophageal wall
Clip 5mm	A 5-mm isotropic expansion around the metallic clip
Clip 5mm in esowall	Intersection between the 5-mm clip expansion and the esophageal wall
GTV with clip	Intersection between the 5-mm clip expansion and the GTV
PTV with clip	Intersection between the 5-mm clip expansion and the PTV

Abbreviations: Eso, esophagus; GTV, gross tumor volume; PTV, planning target volume.

Supplementary Table S2. Paired comparison of dose parameters between native and override plans for photon and proton therapy

Structure/Parameter	Photon plans				Proton plans			
	Native	Override	Δ (95% CI)	p	Native	Override	Δ (95% CI)	p
**Target Volume**								
PTV D95 (%)	101.28 ± 1.42	101.39 ± 1.43	0.11 (-0.05 to 0.26)	0.160	90.86 ± 3.95	90.94 ± 3.96	0.08 (0.04 to 0.12)	< 0.001*
PTV Dmax (Gy)	61.60 ± 8.66	61.55 ± 8.69	-0.05 (-0.21 to 0.11)	0.518	61.56 ± 8.52	61.57 ± 8.54	0.01 (-0.03 to 0.06)	0.556
PTV with Clip D95 (Gy)	55.89 ± 7.34	56.23 ± 7.47	0.35 (0.15 to 0.55)	0.001*	55.12 ± 7.50	55.17 ± 7.48	0.05 (-0.04 to 0.14)	0.247
PTV with Clip Dmax (Gy)	59.20 ± 8.17	59.47 ± 8.04	0.27 (-0.08 to 0.62)	0.126	59.58 ± 8.10	59.60 ± 8.14	0.02 (-0.04 to 0.07)	0.522
GTV D95 (%)	102.35 ± 1.67	102.51 ± 1.67	0.16 (0.01 to 0.31)	0.038*	101.06 ± 0.64	101.10 ± 0.64	0.03 (0.00 to 0.06)	0.029*
GTV Dmax (Gy)	59.73 ± 8.16	59.75 ± 8.18	0.03 (-0.13 to 0.18)	0.725	60.26 ± 8.06	60.32 ± 8.08	0.05 (-0.05 to 0.16)	0.310
GTV with Clip D95 (Gy)	55.88 ± 7.35	56.31 ± 7.54	0.44 (0.20 to 0.68)	< 0.001*	55.32 ± 7.59	55.40 ± 7.60	0.08 (0.02 to 0.13)	0.007*
GTV with Clip Dmax (Gy)	59.35 ± 8.00	59.44 ± 8.03	0.10 (-0.11 to 0.31)	0.347	59.25 ± 7.94	59.41 ± 8.09	0.16 (-0.05 to 0.36)	0.137
**Metallic Clip Region**								
Clip Dmean (Gy)	56.19 ± 7.30	57.28 ± 7.57	1.09 (0.67 to 1.50)	< 0.001*	56.30 ± 7.68	56.59 ± 7.70	0.28 (0.23 to 0.34)	< 0.001*
Clip Dmax (Gy)	58.10 ± 7.70	58.48 ± 7.74	0.38 (-0.05 to 0.81)	0.080	58.31 ± 7.88	58.46 ± 7.95	0.16 (0.07 to 0.25)	0.001*
Clip D0.1cc (Gy)	56.76 ± 7.35	57.65 ± 7.54	0.89 (0.63 to 1.14)	< 0.001*	56.91 ± 7.70	57.23 ± 7.80	0.32 (0.17 to 0.47)	< 0.001*
Clip 5mm Dmean (Gy)	57.04 ± 7.53	57.16 ± 7.58	0.12 (0.00 to 0.24)	0.049*	56.55 ± 7.65	56.59 ± 7.66	0.04 (0.03 to 0.06)	< 0.001*
Clip 5mm Dmax (Gy)	59.44 ± 8.04	59.48 ± 8.04	0.04 (-0.17 to 0.24)	0.732	59.59 ± 8.11	59.59 ± 8.13	0.00 (-0.06 to 0.06)	0.904
Clip 5mm D0.1cc (Gy)	58.52 ± 7.88	58.42 ± 7.82	-0.10 (-0.46 to 0.26)	0.571	58.64 ± 7.91	58.71 ± 7.99	0.07 (-0.06 to 0.20)	0.268
Clip 5mm in EsoWall Dmean (Gy)	56.98 ± 7.42	57.17 ± 7.59	0.19 (0.01 to 0.38)	0.041*	56.58 ± 7.67	56.63 ± 7.69	0.06 (0.03 to 0.08)	< 0.001*
Clip 5mm in EsoWall Dmax (Gy)	59.12 ± 8.03	59.06 ± 8.06	-0.06 (-0.23 to 0.12)	0.502	59.28 ± 8.12	59.27 ± 8.14	-0.01 (-0.08 to 0.05)	0.642
Clip 5mm in EsoWall D0.1cc (Gy)	58.07 ± 7.80	58.01 ± 7.73	-0.06 (-0.39 to 0.28)	0.734	58.02 ± 7.93	58.11 ± 7.97	0.09 (-0.09 to 0.28)	0.312
Clip 3mm Dmean (Gy)	56.97 ± 7.51	57.22 ± 7.60	0.25 (0.12 to 0.39)	< 0.001*	56.51 ± 7.67	56.60 ± 7.70	0.10 (0.07 to 0.13)	< 0.001*

Clip 3mm Dmax (Gy)	59.32 ± 7.95	59.33 ± 7.99	0.01 (-0.19 to 0.21)	0.924	59.27 ± 8.04	59.23 ± 8.02	-0.04 (-0.14 to 0.06)	0.431
Clip 3mm D0.1cc (Gy)	58.30 ± 7.81	58.38 ± 7.83	0.09 (-0.05 to 0.22)	0.221	58.18 ± 7.87	58.26 ± 7.92	0.09 (-0.02 to 0.19)	0.113
Clip 3mm in EsoWall Dmean (Gy)	56.91 ± 7.53	57.22 ± 7.62	0.31 (0.14 to 0.48)	< 0.001*	56.52 ± 7.62	56.66 ± 7.66	0.14 (0.09 to 0.20)	< 0.001*
Clip 3mm in EsoWall Dmax (Gy)	58.96 ± 8.09	58.93 ± 8.09	-0.02 (-0.19 to 0.14)	0.760	58.79 ± 7.92	58.80 ± 7.96	0.02 (-0.05 to 0.08)	0.640
Clip 3mm in EsoWall D0.1cc (Gy)	57.76 ± 7.76	57.96 ± 7.81	0.20 (0.05 to 0.35)	0.012*	57.34 ± 7.83	57.42 ± 7.85	0.07 (-0.04 to 0.18)	0.184
**Esophagus Region**								
EsoWall Dmean (Gy)	31.44 ± 10.74	31.62 ± 10.85	0.18 (-0.11 to 0.46)	0.209	30.00 ± 11.72	30.00 ± 11.72	0.00 (-0.02 to 0.02)	0.769
EsoWall Dmax (Gy)	60.16 ± 8.79	60.07 ± 8.79	-0.09 (-0.30 to 0.12)	0.374	60.81 ± 8.39	60.78 ± 8.38	-0.03 (-0.11 to 0.04)	0.380
EsoWall V50 (%)	42.81 ± 26.53	42.83 ± 26.55	0.02 (-0.04 to 0.07)	0.477	41.39 ± 26.52	41.39 ± 26.52	-0.00 (-0.03 to 0.03)	1.000
EsoWall V60 (%)	25.42 ± 26.52	25.70 ± 26.61	0.28 (0.04 to 0.52)	0.025*	23.19 ± 25.04	23.20 ± 25.07	0.01 (-0.05 to 0.07)	0.802
EsoWall in PTV Dmean (Gy)	56.94 ± 7.54	57.04 ± 7.60	0.10 (-0.01 to 0.21)	0.070	56.67 ± 7.74	56.57 ± 7.68	-0.10 (-0.33 to 0.13)	0.368
EsoWall in PTV Dmax (Gy)	59.77 ± 8.46	59.67 ± 8.49	-0.11 (-0.32 to 0.11)	0.322	60.69 ± 8.42	60.66 ± 8.41	-0.03 (-0.11 to 0.04)	0.369
EsoWall in PTV D0.1cc (Gy)	58.87 ± 8.27	58.91 ± 8.29	0.04 (-0.10 to 0.17)	0.587	59.60 ± 8.18	59.52 ± 8.16	-0.07 (-0.22 to 0.07)	0.297
EsoWall in PTV V50 (%)	80.83 ± 40.03	80.76 ± 40.17	-0.08 (-0.22 to 0.07)	0.278	80.51 ± 40.07	80.52 ± 40.07	0.01 (-0.01 to 0.02)	0.327
EsoWall in PTV V60 (%)	49.69 ± 48.71	50.34 ± 48.92	0.65 (0.04 to 1.25)	0.038*	45.88 ± 46.32	45.89 ± 46.43	0.01 (-0.10 to 0.12)	0.833
Esophagus Dmean (Gy)	32.66 ± 11.39	32.68 ± 11.28	0.01 (-0.32 to 0.34)	0.934	31.15 ± 12.08	32.18 ± 14.04	1.03 (-1.37 to 3.43)	0.386
Esophagus Dmax (Gy)	60.30 ± 8.80	60.23 ± 8.79	-0.07 (-0.27 to 0.13)	0.503	60.90 ± 8.40	60.86 ± 8.39	-0.04 (-0.12 to 0.03)	0.262
Esophagus V50 (%)	44.61 ± 27.72	44.54 ± 27.54	-0.07 (-0.73 to 0.60)	0.841	43.25 ± 27.47	42.97 ± 27.45	-0.27 (-0.84 to 0.29)	0.327
Esophagus V60 (%)	26.77 ± 27.61	26.75 ± 27.52	-0.02 (-0.62 to 0.58)	0.948	24.36 ± 26.17	24.40 ± 26.18	0.04 (-0.00 to 0.08)	0.076

Δ indicates the dose difference between override and native plans (override – native). Data are presented as mean ± SD. p-values were obtained using paired t-tests.

Abbreviations: Gy, gray; D95, dose to 95% of the volume; Dmean, mean dose; Dmax, maximum dose; D0.1cc, minimum dose delivered to the hottest 0.1 cc; EsoWall, esophageal wall.

* indicate statistical significance ($p < 0.05$)

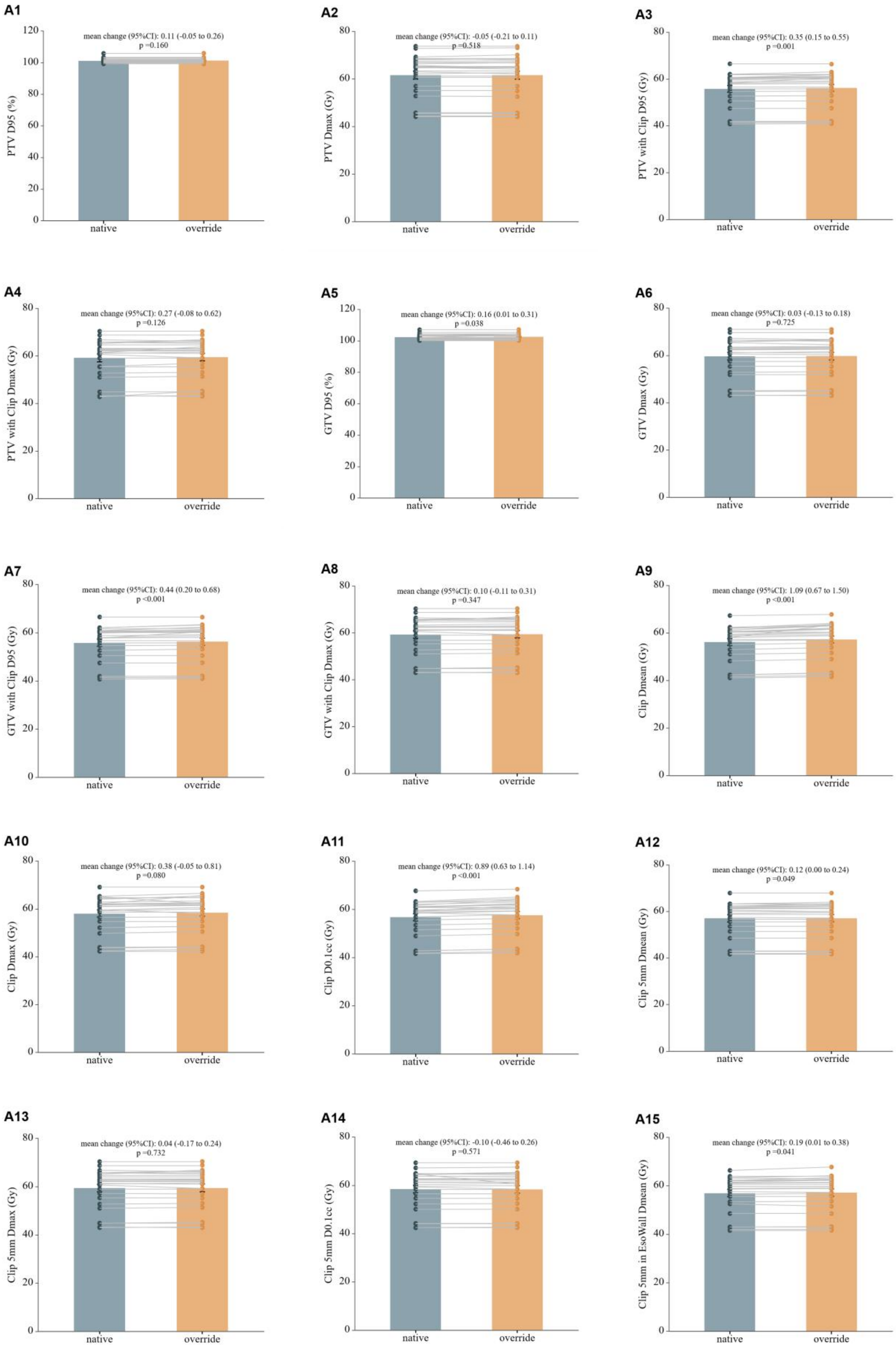
A1
PTV D95 (%)
mean change (95%CI): 0.11 (-0.05 to 0.26)
p =0.160
A2
PTV Dmax (Gy)
mean change (95%CI): -0.05 (-0.21 to 0.11)
p =0.518
A3
PTV with Clip D95 (Gy)
mean change (95%CI): 0.35 (0.15 to 0.55)
p =0.001
A4
PTV with Clip Dmax (Gy)
mean change (95%CI): 0.27 (-0.08 to 0.62)
p =0.126
A5
GTV D95 (%)
mean change (95%CI): 0.16 (0.01 to 0.31)
p =0.038
A6
GTV Dmax (Gy)
mean change (95%CI): 0.03 (-0.13 to 0.18)
p =0.725
A7
GTV with Clip D95 (Gy)
mean change (95%CI): 0.44 (0.20 to 0.68)
p <0.001
A8
GTV with Clip Dmax (Gy)
mean change (95%CI): 0.10 (-0.11 to 0.31)
p =0.347
A9
Clip Dmean (Gy)
mean change (95%CI): 1.09 (0.67 to 1.50)
p <0.001
A10
Clip Dmax (Gy)
mean change (95%CI): 0.38 (-0.05 to 0.81)
p =0.080
A11
Clip D0.1cc (Gy)
mean change (95%CI): 0.89 (0.63 to 1.14)
p <0.001
A12
Clip 5mm Dmean (Gy)
mean change (95%CI): 0.12 (0.00 to 0.24)
p =0.049
A13
Clip 5mm Dmax (Gy)
mean change (95%CI): 0.04 (-0.17 to 0.24)
p =0.732
A14
Clip 5mm D0.1cc (Gy)
mean change (95%CI): -0.10 (-0.46 to 0.26)
p =0.571
A15
Clip 5mm in EsoWall Dmean (Gy)
mean change (95%CI): 0.19 (0.01 to 0.38)
p =0.041
native
override

A16

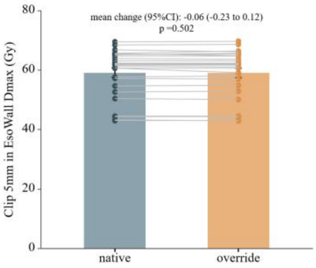

A17

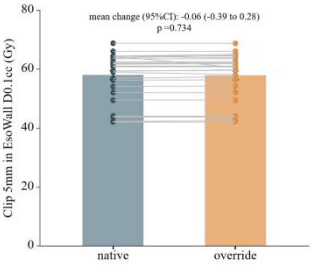

A18

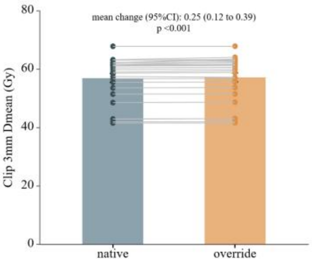

A19

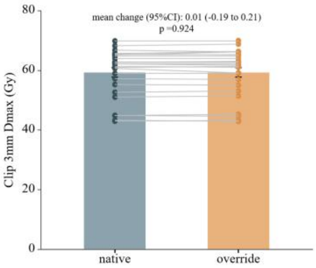

A20

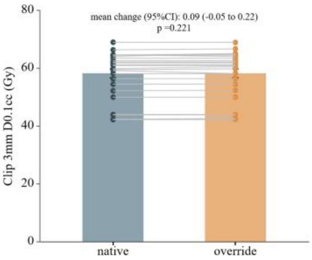

A21

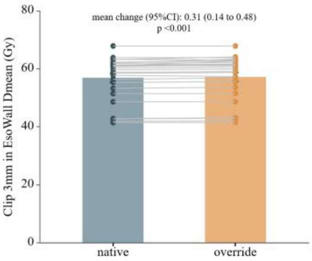

A22

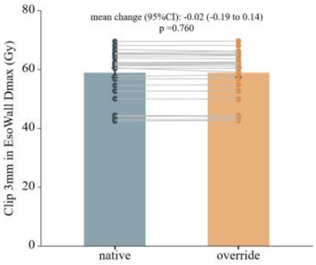

A23

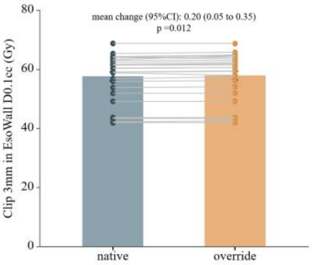

A24

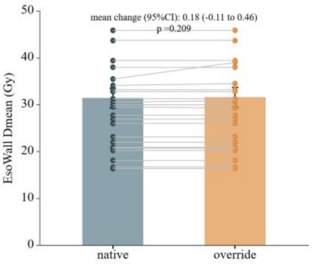

A25

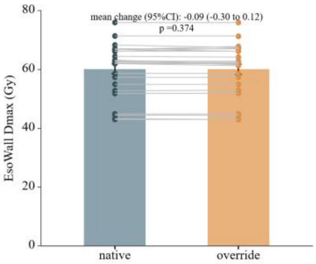

A26

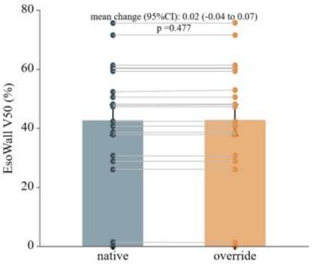

A27

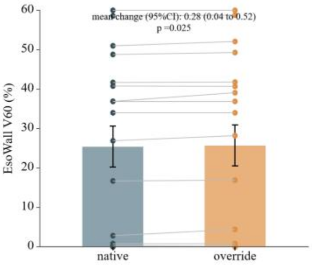

A28

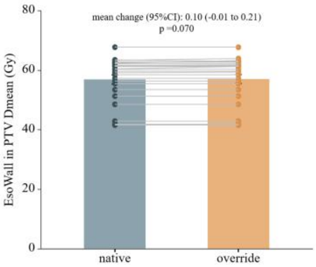

A29

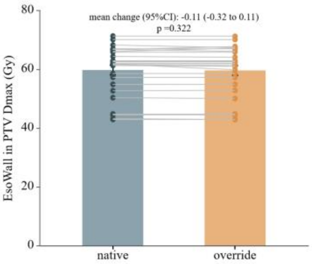

A30

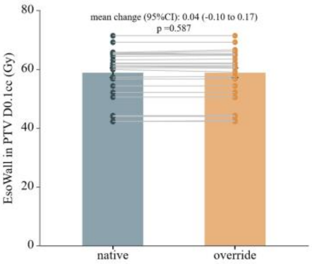

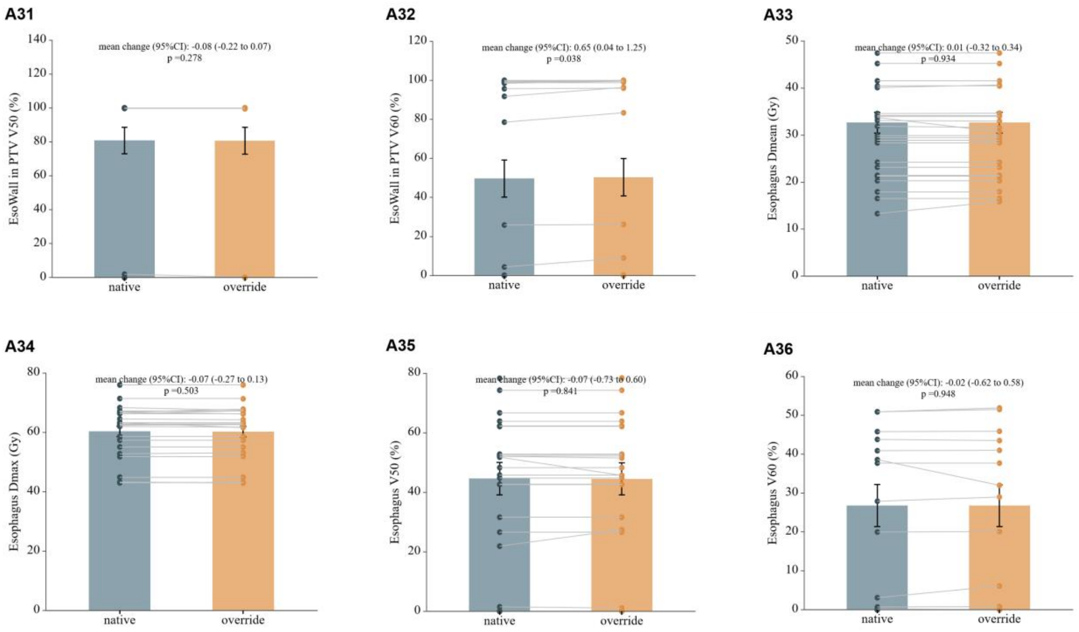

Supplementary Figure S1.Paired comparisons between native and override plans for proton therapy (Panels A1–A36).

Each bar represents the mean±SD of dose differences, with individual patient values shown as dots. Mean change (95% confidence interval) and p-values were derived from paired t-tests.

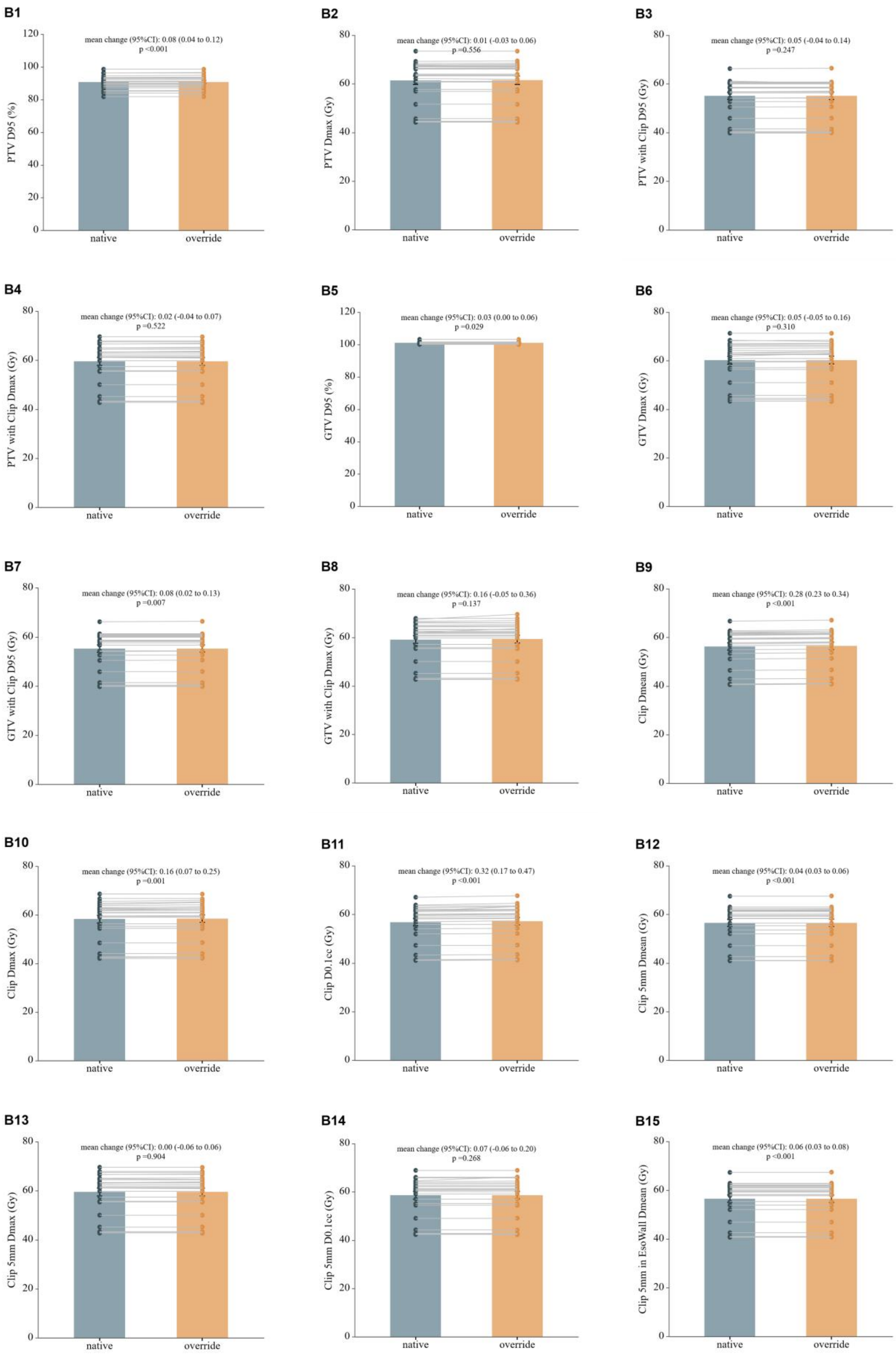
B1
mean change (95%CI): 0.08 (0.04 to 0.12)
p <0.001
PTV D95 (%)
native
override
B2
mean change (95%CI): 0.01 (-0.03 to 0.06)
p =0.556
PTV Dmax (Gy)
native
override
B3
mean change (95%CI): 0.05 (-0.04 to 0.14)
p =0.247
PTV with Clip D95 (Gy)
native
override
B4
mean change (95%CI): 0.02 (-0.04 to 0.07)
p =0.522
PTV with Clip Dmax (Gy)
native
override
B5
mean change (95%CI): 0.03 (0.00 to 0.06)
p =0.029
GTV D95 (%)
native
override
B6
mean change (95%CI): 0.05 (-0.05 to 0.16)
p =0.310
GTV Dmax (Gy)
native
override
B7
mean change (95%CI): 0.08 (0.02 to 0.13)
p =0.007
GTV with Clip D95 (Gy)
native
override
B8
mean change (95%CI): 0.16 (-0.05 to 0.36)
p =0.137
GTV with Clip Dmax (Gy)
native
override
B9
mean change (95%CI): 0.28 (0.23 to 0.34)
p <0.001
Clip Dmean (Gy)
native
override
B10
mean change (95%CI): 0.16 (0.07 to 0.25)
p =0.001
Clip Dmax (Gy)
native
override
B11
mean change (95%CI): 0.32 (0.17 to 0.47)
p <0.001
Clip D0.1cc (Gy)
native
override
B12
mean change (95%CI): 0.04 (0.03 to 0.06)
p <0.001
Clip 5mm Dmean (Gy)
native
override
B13
mean change (95%CI): 0.00 (-0.06 to 0.06)
p =0.904
Clip 5mm Dmax (Gy)
native
override
B14
mean change (95%CI): 0.07 (-0.06 to 0.20)
p =0.268
Clip 5mm D0.1cc (Gy)
native
override
B15
mean change (95%CI): 0.06 (0.03 to 0.08)
p <0.001
Clip 5mm in EsoWall Dmean (Gy)
native
override

B16

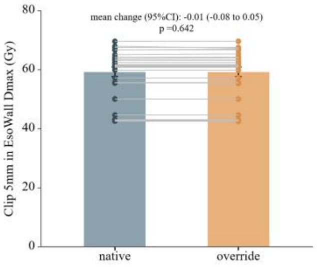

B17

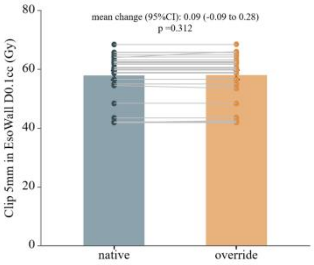

B18

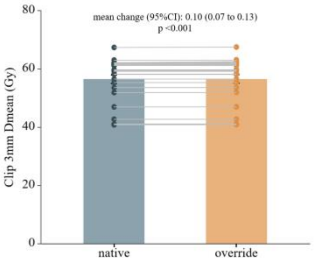

B19

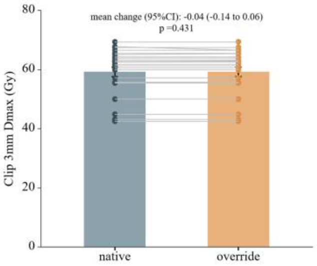

B20

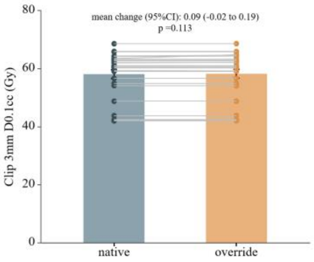

B21

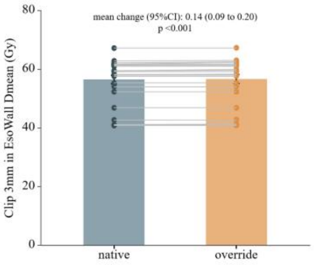

B22

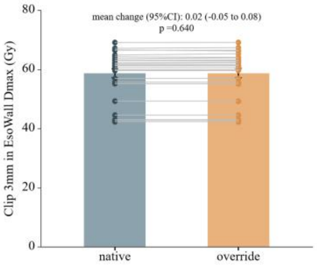

B23

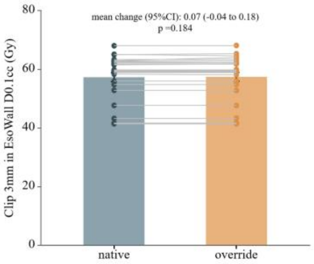

B24

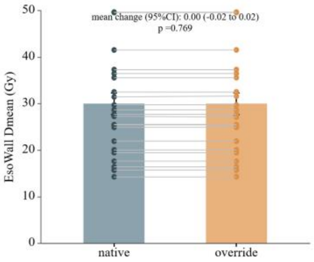

B25

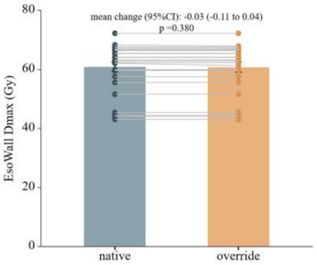

B26

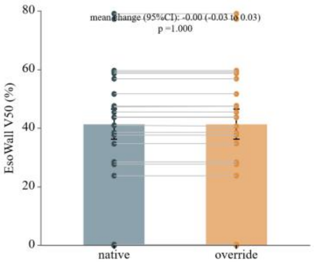

B27

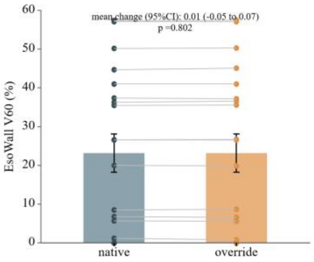

B28

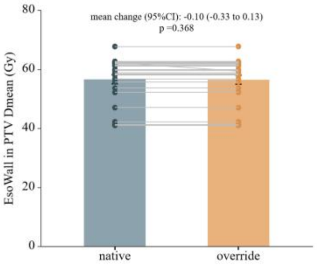

B29

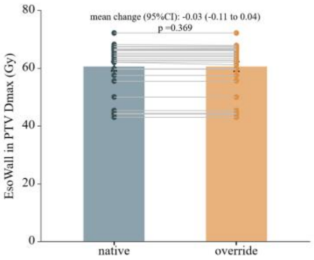

B30

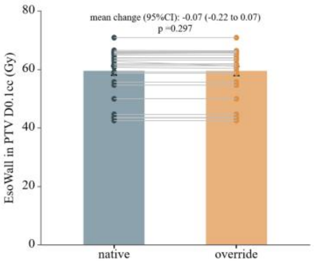

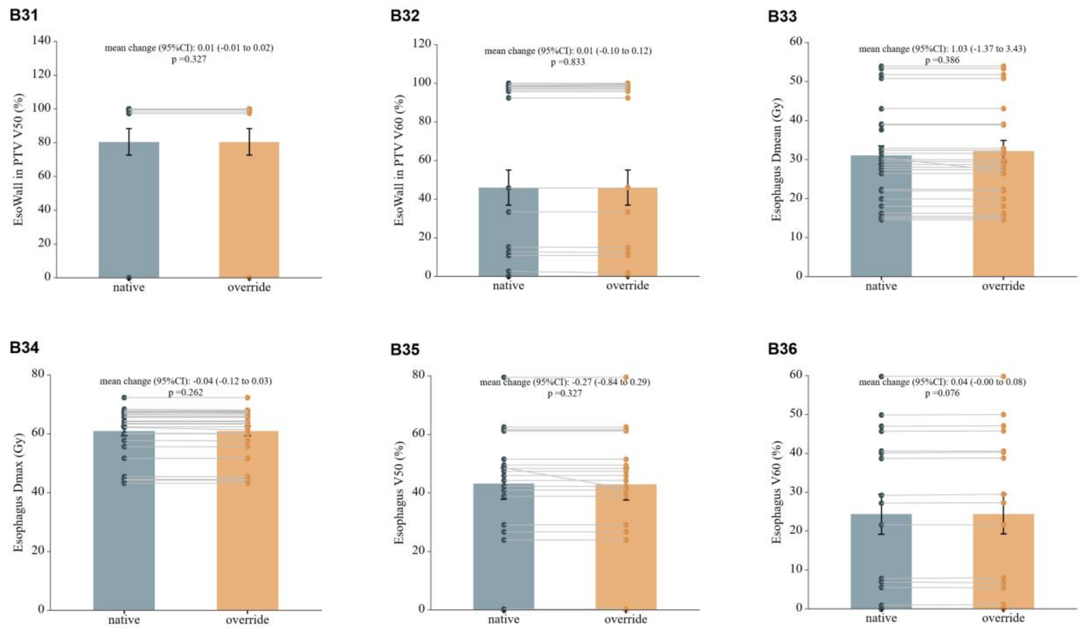

Supplementary Figure S2. Paired comparisons between native and override plans for proton therapy (Panels B1–B36).

Each bar represents the mean±SD of dose differences, with individual patient values shown as dots. Mean change (95% confidence interval) and p-values were derived from paired t-tests.

Supplementary Table S3. Comparison of dose perturbation between photon and proton therapy plans after CT value override of metallic clips

Structure/Parameters	Photon		Proton		Difference (95% CI) (Gy)	Difference (95% CI) (%)	p (Gy)	p (%)
	Δ (Gy)	Δ%	Δ (Gy)	Δ%				
**Target Volume**								
PTV D95 (%)	0.11 ± 0.38	0.11 ± 0.38	0.08 ± 0.10	0.08 ± 0.11	-0.03 (-0.18 to 0.12)	-0.02 (-0.17 to 0.13)	0.677	0.760
PTV Dmax (Gy)	-0.05 ± 0.39	-0.09 ± 0.59	0.01 ± 0.12	0.02 ± 0.17	0.06 (-0.09 to 0.22)	0.10 (-0.13 to 0.33)	0.398	0.364
PTV with Clip D95 (Gy)	0.35 ± 0.49	0.60 ± 0.82	0.05 ± 0.21	0.10 ± 0.36	-0.30 (-0.54 to -0.05)	-0.50 (-0.91 to -0.10)	0.019*	0.017*
PTV with Clip Dmax (Gy)	0.27 ± 0.88	0.52 ± 1.63	0.02 ± 0.14	0.02 ± 0.22	-0.25 (-0.62 to 0.12)	-0.49 (-1.18 to 0.19)	0.169	0.149
GTV D95 (%)	0.16 ± 0.38	0.16 ± 0.37	0.03 ± 0.07	0.03 ± 0.07	-0.13 (-0.28 to 0.02)	-0.13 (-0.28 to 0.02)	0.092	0.091
GTV Dmax (Gy)	0.03 ± 0.37	0.04 ± 0.59	0.05 ± 0.26	0.08 ± 0.41	0.03 (-0.16 to 0.21)	0.04 (-0.25 to 0.33)	0.768	0.778
GTV with Clip D95 (Gy)	0.44 ± 0.59	0.75 ± 1.00	0.08 ± 0.13	0.14 ± 0.23	-0.36 (-0.61 to -0.11)	-0.61 (-1.03 to -0.19)	0.006*	0.006*
GTV with Clip Dmax (Gy)	0.10 ± 0.52	0.16 ± 0.85	0.15 ± 0.52	0.24 ± 0.79	0.06 (-0.29 to 0.40)	0.07 (-0.48 to 0.63)	0.735	0.794
**Metallic Clip Region**								
Clip Dmean (Gy)	1.09 ± 1.02	1.92 ± 1.72	0.28 ± 0.13	0.51 ± 0.22	-0.80 (-1.22 to -0.39)	-1.41 (-2.10 to -0.71)	< 0.001*	< 0.001*
Clip Dmax (Gy)	0.38 ± 1.06	0.67 ± 1.70	0.15 ± 0.22	0.26 ± 0.35	-0.22 (-0.63 to 0.18)	-0.41 (-1.07 to 0.24)	0.266	0.203
Clip D0.1cc (Gy)	0.89 ± 0.63	1.55 ± 1.03	0.32 ± 0.37	0.54 ± 0.59	-0.57 (-0.85 to -0.30)	-1.01 (-1.47 to -0.56)	< 0.001*	< 0.001*
Clip 5mm Dmean (Gy)	0.12 ± 0.30	0.20 ± 0.48	0.04 ± 0.04	0.07 ± 0.07	-0.08 (-0.20 to 0.04)	-0.13 (-0.32 to 0.07)	0.199	0.185
Clip 5mm Dmax (Gy)	0.04 ± 0.51	0.06 ± 0.84	0.00 ± 0.15	-0.00 ± 0.23	-0.03 (-0.26 to 0.20)	-0.06 (-0.44 to 0.31)	0.781	0.728
Clip 5mm D0.1cc (Gy)	-0.10 ± 0.90	-0.15 ± 1.40	0.07 ± 0.31	0.10 ± 0.48	0.17 (-0.21 to 0.55)	0.25 (-0.34 to 0.84)	0.365	0.393
Clip 5mm in EsoWall Dmean (Gy)	0.19 ± 0.46	0.31 ± 0.75	0.06 ± 0.05	0.10 ± 0.09	-0.14 (-0.32 to 0.04)	-0.21 (-0.51 to 0.08)	0.126	0.153
Clip 5mm in EsoWall Dmax (Gy)	-0.06 ± 0.43	-0.10 ± 0.70	-0.01 ± 0.16	-0.03 ± 0.25	0.04 (-0.13 to 0.21)	0.07 (-0.21 to 0.35)	0.608	0.605
Clip 5mm in EsoWall D0.1cc (Gy)	-0.06 ± 0.83	-0.07 ± 1.30	0.09 ± 0.46	0.16 ± 0.78	0.15 (-0.23 to 0.53)	0.23 (-0.37 to 0.84)	0.426	0.439
Clip 3mm Dmean (Gy)	0.25 ± 0.33	0.43 ± 0.52	0.10 ± 0.07	0.17 ± 0.12	-0.15 (-0.30 to -0.01)	-0.26 (-0.49 to -0.02)	0.039*	0.032*

Clip 3mm Dmax (Gy)	0.01 ± 0.49	0.01 ± 0.81	-0.04 ± 0.24	-0.06 ± 0.36	-0.05 (-0.25 to 0.16)	-0.07 (-0.41 to 0.26)	0.633	0.651
Clip 3mm D0.1cc (Gy)	0.08 ± 0.34	0.14 ± 0.57	0.09 ± 0.27	0.14 ± 0.42	0.00 (-0.18 to 0.18)	-0.01 (-0.30 to 0.29)	0.990	0.966
Clip 3mm in EsoWall Dmean (Gy)	0.31 ± 0.42	0.54 ± 0.68	0.14 ± 0.14	0.25 ± 0.22	-0.16 (-0.35 to 0.02)	-0.28 (-0.58 to 0.01)	0.075	0.061
Clip 3mm in EsoWall Dmax (Gy)	-0.02 ± 0.41	-0.04 ± 0.66	0.01 ± 0.17	0.02 ± 0.26	0.04 (-0.12 to 0.20)	0.06 (-0.20 to 0.31)	0.604	0.659
Clip 3mm in EsoWall D0.1cc (Gy)	0.20 ± 0.37	0.34 ± 0.61	0.07 ± 0.27	0.13 ± 0.43	-0.13 (-0.30 to 0.05)	-0.22 (-0.50 to 0.07)	0.156	0.137
**Esophagus Region**								
EsoWall Dmean (Gy)	0.18 ± 0.70	0.49 ± 1.97	0.00 ± 0.05	0.00 ± 0.16	-0.17 (-0.45 to 0.11)	-0.49 (-1.28 to 0.30)	0.214	0.216
EsoWall Dmax (Gy)	-0.09 ± 0.51	-0.15 ± 0.80	-0.03 ± 0.19	-0.05 ± 0.30	0.06 (-0.16 to 0.27)	0.10 (-0.24 to 0.43)	0.583	0.559
EsoWall V50 (%)	0.02 ± 0.14	-0.54 ± 2.87	0.00 ± 0.08	-0.01 ± 0.21	-0.02 (-0.08 to 0.04)	0.54 (-0.74 to 1.81)	0.518	0.391
EsoWall V60 (%)	0.28 ± 0.59	0.38 ± 22.04	0.01 ± 0.15	-2.01 ± 8.15	-0.27 (-0.54 to 0.00)	-2.38 (-15.14 to 10.38)	0.052	0.698
EsoWall in PTV Dmean (Gy)	0.10 ± 0.27	0.17 ± 0.44	-0.10 ± 0.56	-0.16 ± 0.90	-0.20 (-0.40 to 0.00)	-0.33 (-0.65 to -0.00)	0.050*	0.049*
EsoWall in PTV Dmax (Gy)	-0.11 ± 0.54	-0.18 ± 0.85	-0.03 ± 0.19	-0.06 ± 0.30	0.07 (-0.15 to 0.30)	0.13 (-0.23 to 0.49)	0.509	0.462
EsoWall in PTV D0.1cc (Gy)	0.04 ± 0.34	0.06 ± 0.54	-0.07 ± 0.36	-0.12 ± 0.57	-0.11 (-0.25 to 0.03)	-0.18 (-0.39 to 0.03)	0.108	0.090
EsoWall in PTV V50 (%)	-0.08 ± 0.35	-0.01 ± 0.04	0.01 ± 0.04	0.01 ± 0.04	0.08 (-0.06 to 0.23)	0.02 (-0.02 to 0.06)	0.240	0.329
EsoWall in PTV V60 (%)	0.65 ± 1.50	4.08 ± 38.09	0.01 ± 0.28	-2.12 ± 8.42	-0.63 (-1.30 to 0.03)	-6.20 (-26.65 to 14.25)	0.062	0.529
Esophagus Dmean (Gy)	0.01 ± 0.82	0.46 ± 4.26	1.03 ± 5.95	2.66 ± 15.85	1.02 (-1.38 to 3.41)	2.20 (-4.36 to 8.76)	0.391	0.497
Esophagus Dmax (Gy)	-0.07 ± 0.50	-0.11 ± 0.78	-0.04 ± 0.18	-0.07 ± 0.29	0.03 (-0.19 to 0.24)	0.04 (-0.29 to 0.37)	0.810	0.803
Esophagus V50 (%)	-0.06 ± 1.65	-0.67 ± 8.37	-0.27 ± 1.39	-0.66 ± 3.12	-0.21 (-0.66 to 0.24)	0.01 (-3.55 to 3.56)	0.348	0.997
Esophagus V60 (%)	-0.02 ± 1.49	0.12 ± 35.25	0.04 ± 0.11	1.34 ± 5.50	0.06 (-0.56 to 0.68)	1.22 (-17.96 to 20.39)	0.850	0.895

Δ represents the mean paired difference between override and native plans (override – native); Δ% the relative percentage difference. Difference (95% CI) represents the mean difference between photon and proton Δ values.

Separate p values are reported for absolute (Gy) and relative (%) differences.

* indicate statistical significance ($p < 0.05$)

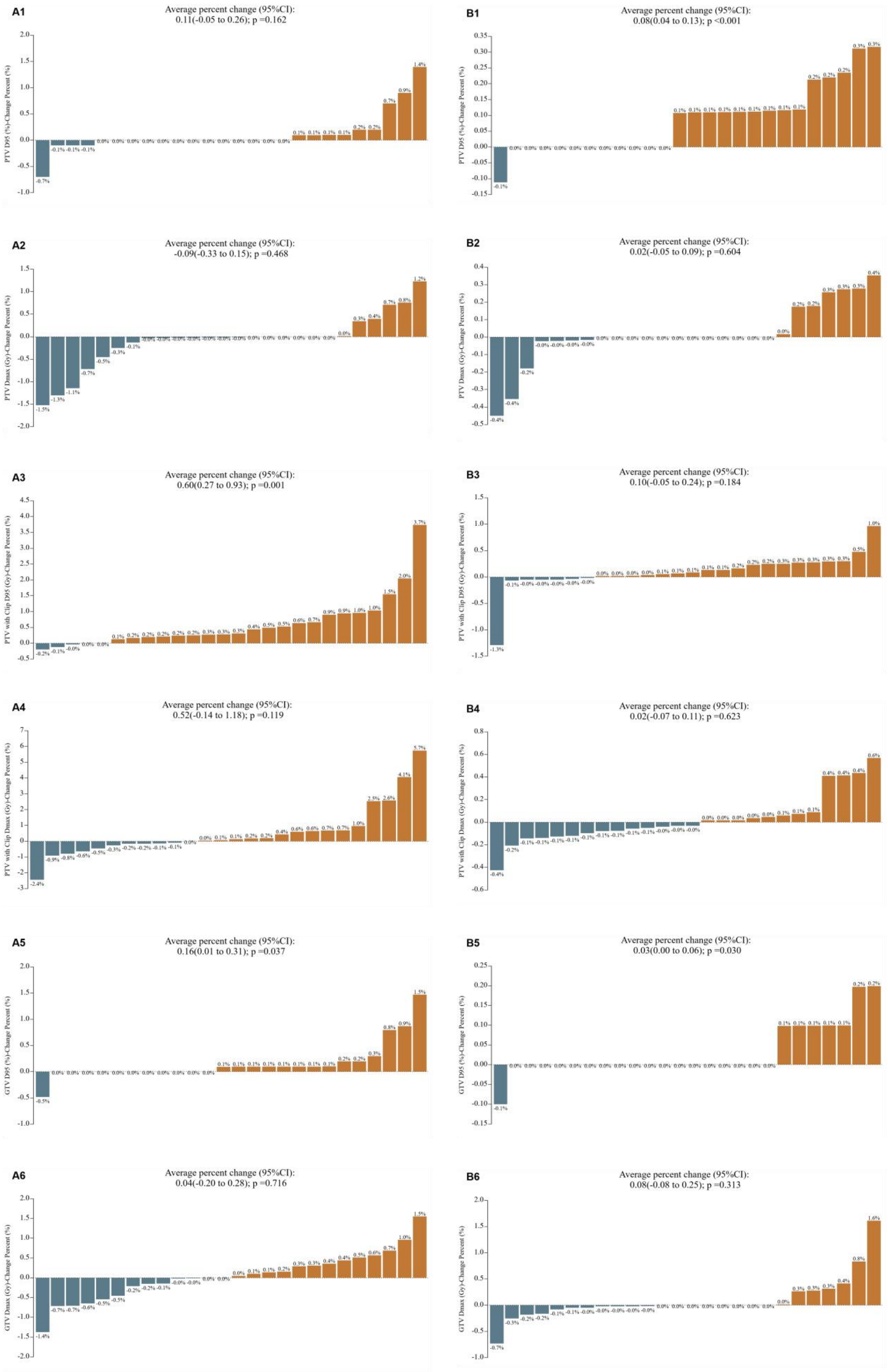
A1
Average percent change (95%CI):
0.11(-0.05 to 0.26); p =0.162
PTV D95 (%)-Change Percent (%)
B1
Average percent change (95%CI):
0.08(0.04 to 0.13); p <0.001
PTV D95 (%)-Change Percent (%)
A2
Average percent change (95%CI):
-0.09(-0.33 to 0.15); p =0.468
PTV Dmax (Gy)-Change Percent (%)
B2
Average percent change (95%CI):
0.02(-0.05 to 0.09); p =0.604
PTV Dmax (Gy)-Change Percent (%)
A3
Average percent change (95%CI):
0.60(0.27 to 0.93); p =0.001
PTV with Clip D95 (Gy)-Change Percent (%)
B3
Average percent change (95%CI):
0.10(-0.05 to 0.24); p =0.184
PTV with Clip D95 (Gy)-Change Percent (%)
A4
Average percent change (95%CI):
0.52(-0.14 to 1.18); p =0.119
PTV with Clip Dmax (Gy)-Change Percent (%)
B4
Average percent change (95%CI):
0.02(-0.07 to 0.11); p =0.623
PTV with Clip Dmax (Gy)-Change Percent (%)
A5
Average percent change (95%CI):
0.16(0.01 to 0.31); p =0.037
GTV D95 (%)-Change Percent (%)
B5
Average percent change (95%CI):
0.03(0.00 to 0.06); p =0.030
GTV D95 (%)-Change Percent (%)
A6
Average percent change (95%CI):
0.04(-0.20 to 0.28); p =0.716
GTV Dmax (Gy)-Change Percent (%)
B6
Average percent change (95%CI):
0.08(-0.08 to 0.25); p =0.313
GTV Dmax (Gy)-Change Percent (%)

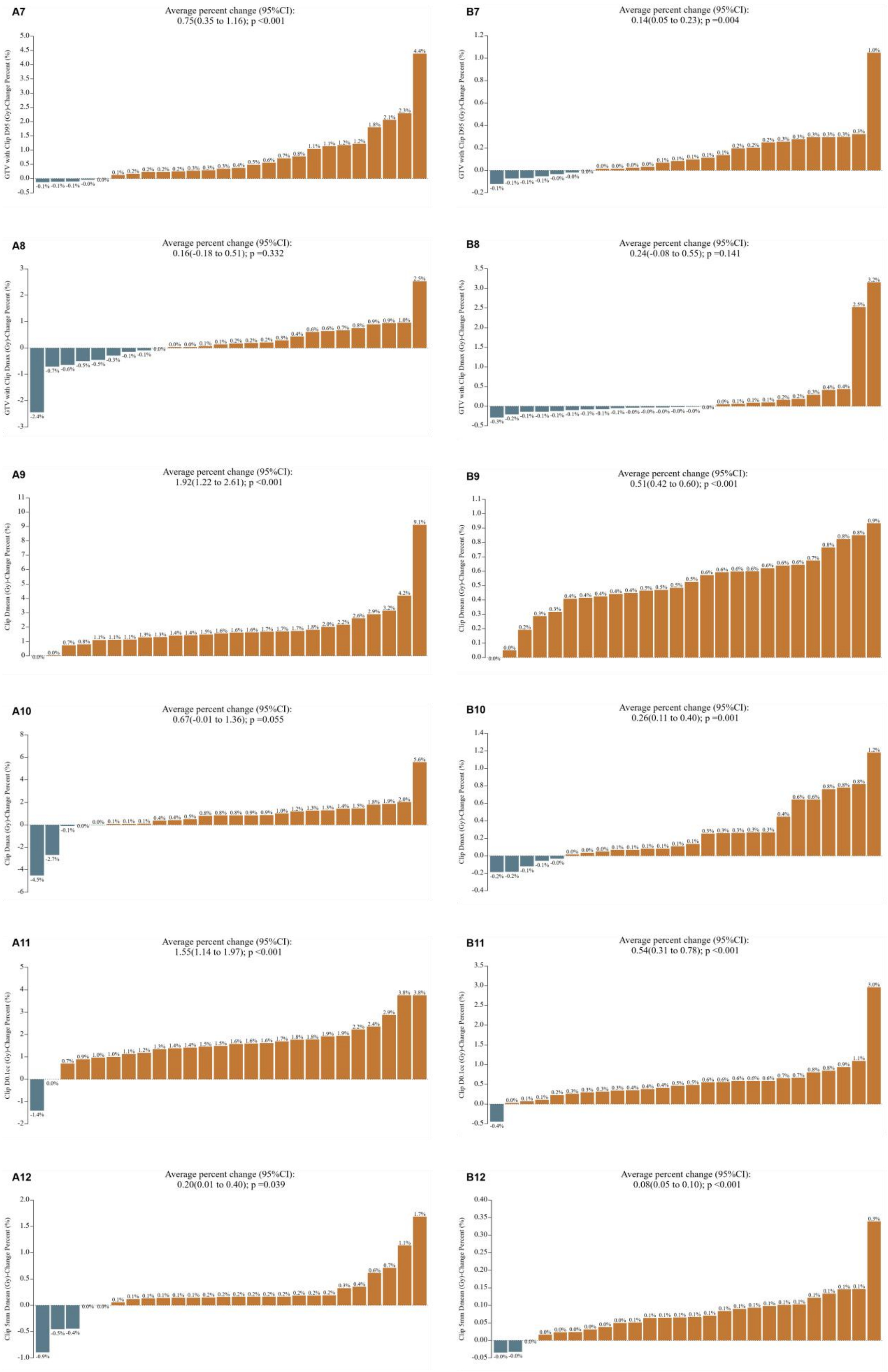
A7
Average percent change (95%CI):
0.75(0.35 to 1.16); p <0.001
GTV with Clip D95 (Gy)-Change Percent (%)
B7
Average percent change (95%CI):
0.14(0.05 to 0.23); p =0.004
GTV with Clip D95 (Gy)-Change Percent (%)
A8
Average percent change (95%CI):
0.16(-0.18 to 0.51); p =0.332
GTV with Clip Dmax (Gy)-Change Percent (%)
B8
Average percent change (95%CI):
0.24(-0.08 to 0.55); p =0.141
GTV with Clip Dmax (Gy)-Change Percent (%)
A9
Average percent change (95%CI):
1.92(1.22 to 2.61); p <0.001
Clip Dmean (Gy)-Change Percent (%)
B9
Average percent change (95%CI):
0.51(0.42 to 0.60); p <0.001
Clip Dmean (Gy)-Change Percent (%)
A10
Average percent change (95%CI):
0.67(-0.01 to 1.36); p =0.055
Clip Dmax (Gy)-Change Percent (%)
B10
Average percent change (95%CI):
0.26(0.11 to 0.40); p =0.001
Clip Dmax (Gy)-Change Percent (%)
A11
Average percent change (95%CI):
1.55(1.14 to 1.97); p <0.001
Clip D0.1cc (Gy)-Change Percent (%)
B11
Average percent change (95%CI):
0.54(0.31 to 0.78); p <0.001
Clip D0.1cc (Gy)-Change Percent (%)
A12
Average percent change (95%CI):
0.20(0.01 to 0.40); p =0.039
Clip 5mm Dmean (Gy)-Change Percent (%)
B12
Average percent change (95%CI):
0.08(0.05 to 0.10); p <0.001
Clip 5mm Dmean (Gy)-Change Percent (%)

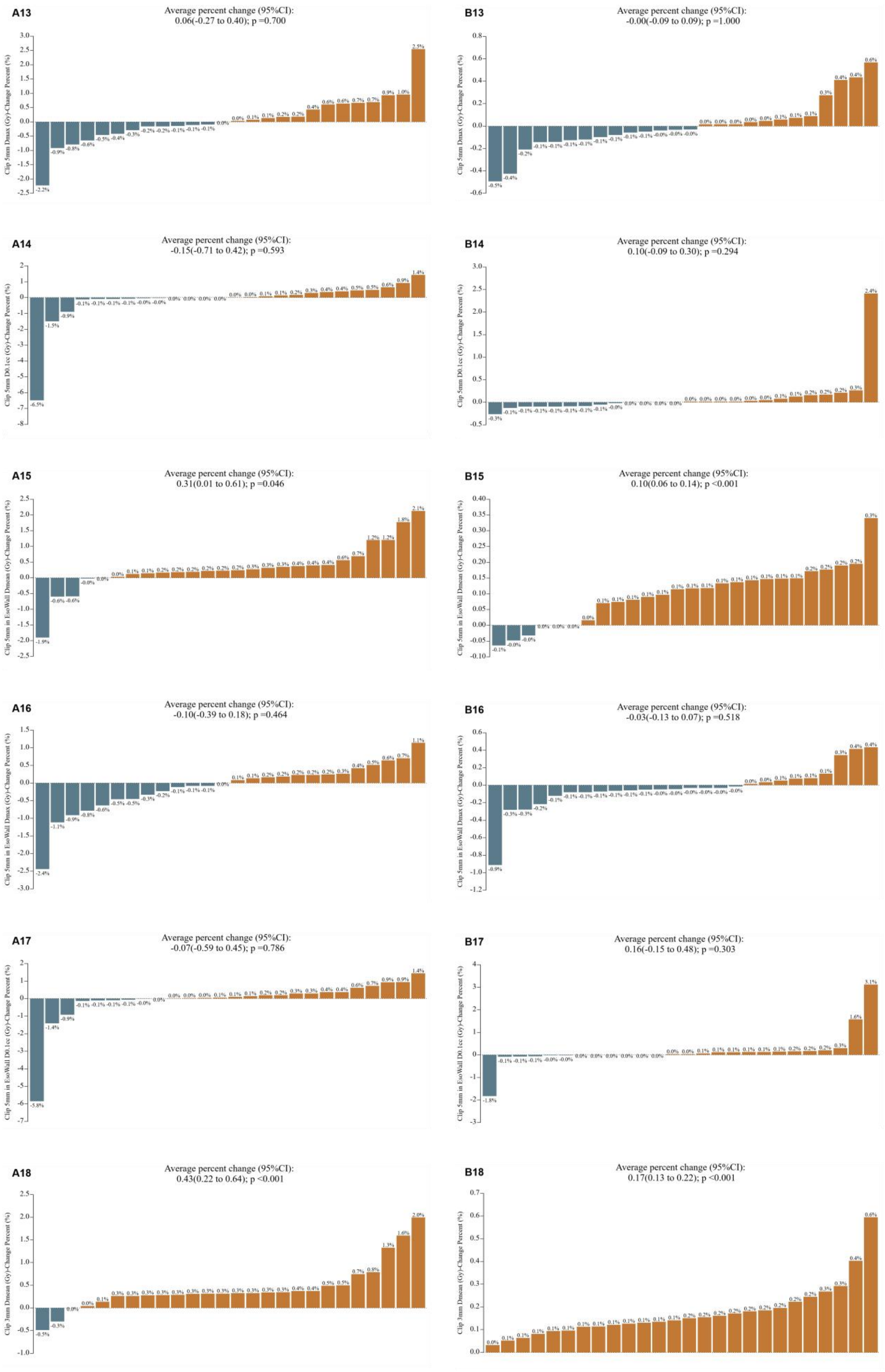
A13
Average percent change (95%CI):
0.06(-0.27 to 0.40); p =0.700
Clip 5mm Dmax (Gy)-Change Percent (%)
B13
Average percent change (95%CI):
-0.00(-0.09 to 0.09); p =1.000
Clip 5mm Dmax (Gy)-Change Percent (%)
A14
Average percent change (95%CI):
-0.15(-0.71 to 0.42); p =0.593
Clip 5mm D0.1cc (Gy)-Change Percent (%)
B14
Average percent change (95%CI):
0.10(-0.09 to 0.30); p =0.294
Clip 5mm D0.1cc (Gy)-Change Percent (%)
A15
Average percent change (95%CI):
0.31(0.01 to 0.61); p =0.046
Clip 5mm in EsoWall Dmean (Gy)-Change Percent (%)
B15
Average percent change (95%CI):
0.10(0.06 to 0.14); p <0.001
Clip 5mm in EsoWall Dmean (Gy)-Change Percent (%)
A16
Average percent change (95%CI):
-0.10(-0.39 to 0.18); p =0.464
Clip 5mm in EsoWall Dmax (Gy)-Change Percent (%)
B16
Average percent change (95%CI):
-0.03(-0.13 to 0.07); p =0.518
Clip 5mm in EsoWall Dmax (Gy)-Change Percent (%)
A17
Average percent change (95%CI):
-0.07(-0.59 to 0.45); p =0.786
Clip 5mm in EsoWall D0.1cc (Gy)-Change Percent (%)
B17
Average percent change (95%CI):
0.16(-0.15 to 0.48); p =0.303
Clip 5mm in EsoWall D0.1cc (Gy)-Change Percent (%)
A18
Average percent change (95%CI):
0.43(0.22 to 0.64); p <0.001
Clip 3mm Dmean (Gy)-Change Percent (%)
B18
Average percent change (95%CI):
0.17(0.13 to 0.22); p <0.001
Clip 3mm Dmean (Gy)-Change Percent (%)

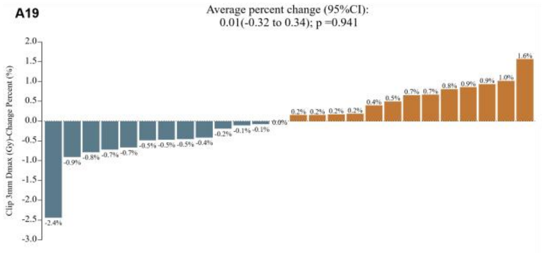
A19
Average percent change (95%CI):
0.01(-0.32 to 0.34); p =0.941
Clip 3mm Dmax (Gy)-Change Percent (%)

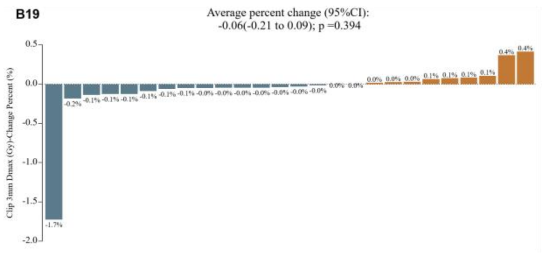
B19
Average percent change (95%CI):
-0.06(-0.21 to 0.09); p =0.394
Clip 3mm Dmax (Gy)-Change Percent (%)

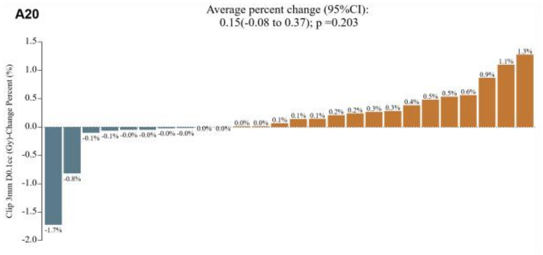
A20
Average percent change (95%CI):
0.15(-0.08 to 0.37); p =0.203
Clip 3mm D0.1cc (Gy)-Change Percent (%)

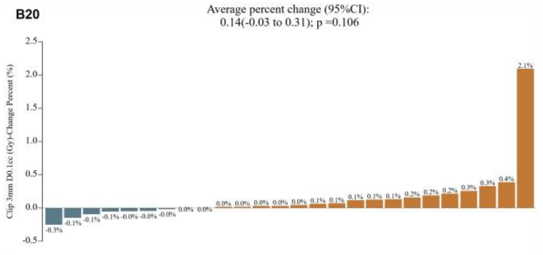
B20
Average percent change (95%CI):
0.14(-0.03 to 0.31); p =0.106
Clip 3mm D0.1cc (Gy)-Change Percent (%)

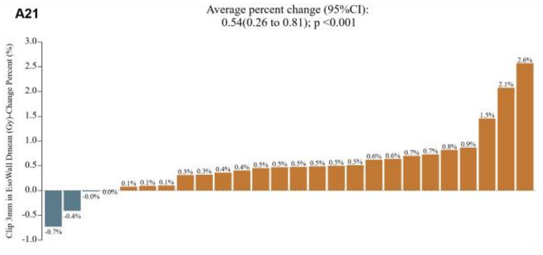
A21
Average percent change (95%CI):
0.54(0.26 to 0.81); p <0.001
Clip 3mm in EsoWall Dmean (Gy)-Change Percent (%)

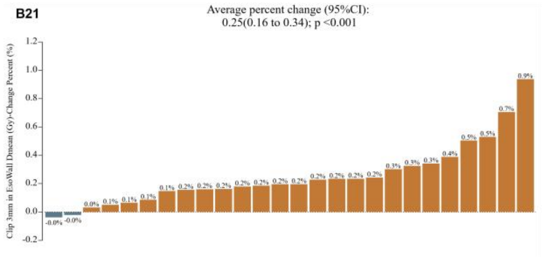
B21
Average percent change (95%CI):
0.25(0.16 to 0.34); p <0.001
Clip 3mm in EsoWall Dmean (Gy)-Change Percent (%)

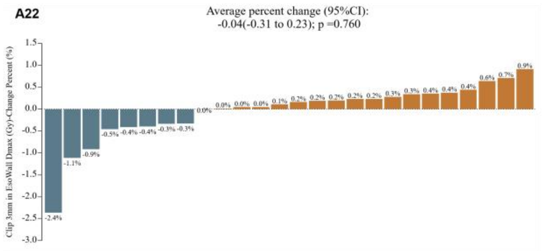
A22
Average percent change (95%CI):
-0.04(-0.31 to 0.23); p =0.760
Clip 3mm in EsoWall Dmax (Gy)-Change Percent (%)

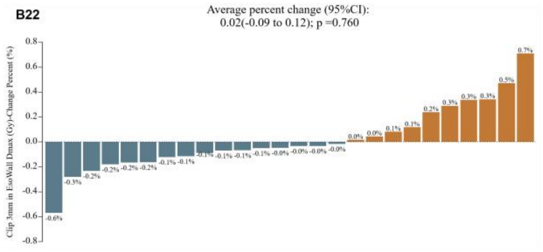
B22
Average percent change (95%CI):
0.02(-0.09 to 0.12); p =0.760
Clip 3mm in EsoWall Dmax (Gy)-Change Percent (%)

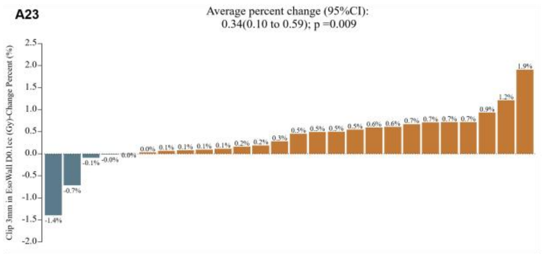
A23
Average percent change (95%CI):
0.34(0.10 to 0.59); p =0.009
Clip 3mm in EsoWall D0.1cc (Gy)-Change Percent (%)

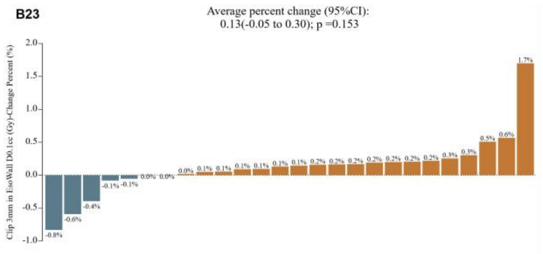
B23
Average percent change (95%CI):
0.13(-0.05 to 0.30); p =0.153
Clip 3mm in EsoWall D0.1cc (Gy)-Change Percent (%)

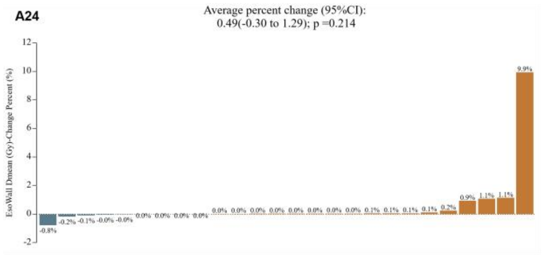
A24
Average percent change (95%CI):
0.49(-0.30 to 1.29); p =0.214
EsoWall Dmean (Gy)-Change Percent (%)

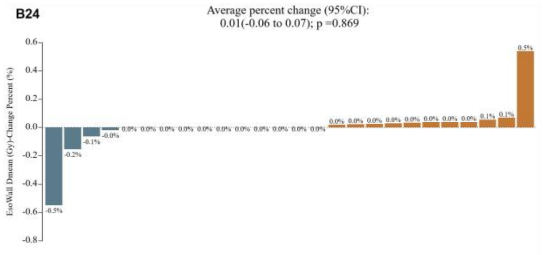
B24
Average percent change (95%CI):
0.01(-0.06 to 0.07); p =0.869
EsoWall Dmean (Gy)-Change Percent (%)

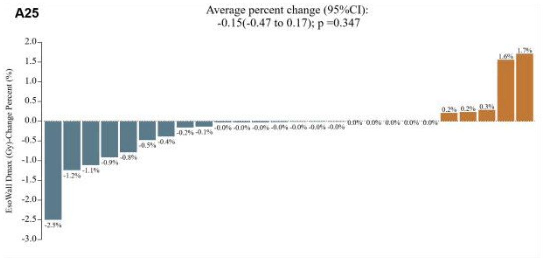
A25
Average percent change (95%CI):
-0.15(-0.47 to 0.17); p =0.347
EsoWall Dmax (Gy)-Change Percent (%)

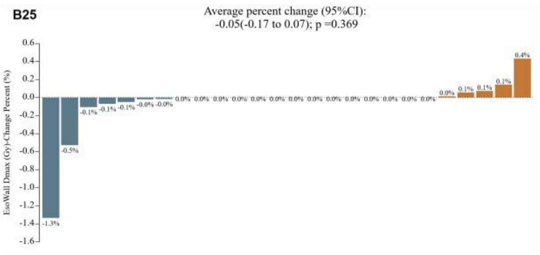
B25
Average percent change (95%CI):
-0.05(-0.17 to 0.07); p =0.369
EsoWall Dmax (Gy)-Change Percent (%)

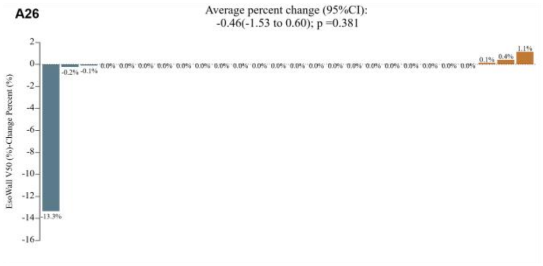
A26
Average percent change (95%CI):
-0.46(-1.53 to 0.60); p =0.381
EsoWall V50 (%)-Change Percent (%)

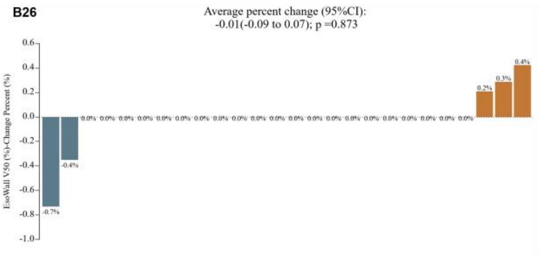
B26
Average percent change (95%CI):
-0.01(-0.09 to 0.07); p =0.873
EsoWall V50 (%)-Change Percent (%)

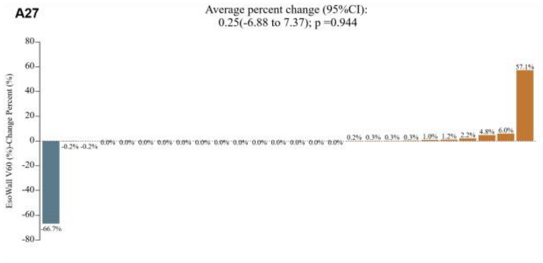
A27
Average percent change (95%CI):
0.25(-6.88 to 7.37); p =0.944
EsoWall V60 (%)-Change Percent (%)

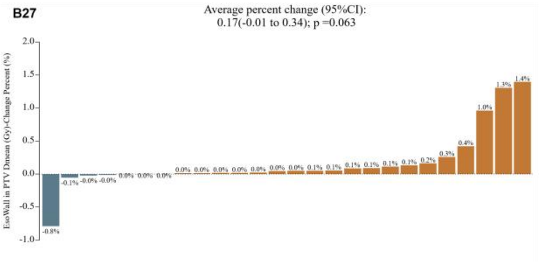
B27
Average percent change (95%CI):
0.17(-0.01 to 0.34); p =0.063
EsoWall in PTV Dmean (Gy)-Change Percent (%)

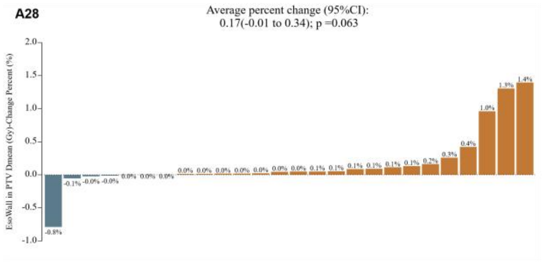
A28
Average percent change (95%CI):
0.17(-0.01 to 0.34); p =0.063
EsoWall in PTV Dmean (Gy)-Change Percent (%)

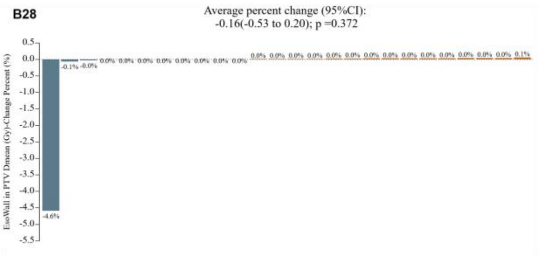
B28
Average percent change (95%CI):
-0.16(-0.53 to 0.20); p =0.372
EsoWall in PTV Dmean (Gy)-Change Percent (%)

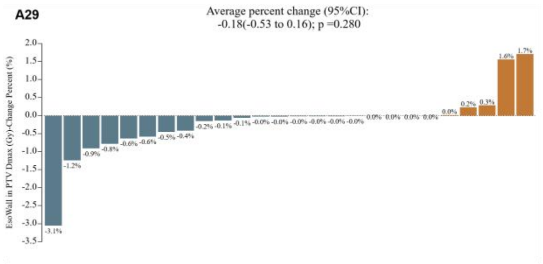
A29
Average percent change (95%CI):
-0.18(-0.53 to 0.16); p =0.280
EsoWall in PTV Dmax (Gy)-Change Percent (%)

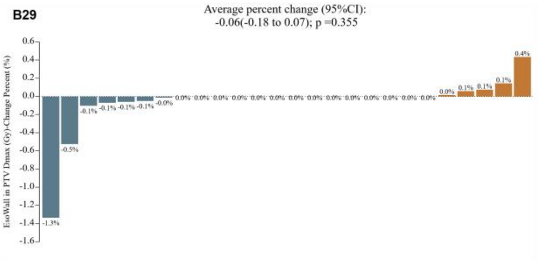
B29
Average percent change (95%CI):
-0.06(-0.18 to 0.07); p =0.355
EsoWall in PTV Dmax (Gy)-Change Percent (%)

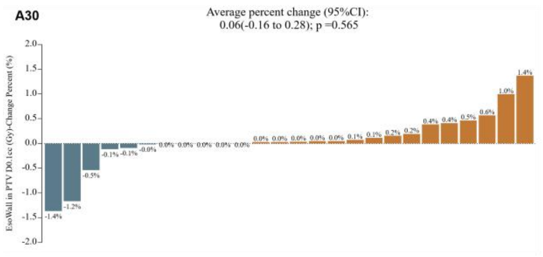
A30
Average percent change (95%CI):
0.06(-0.16 to 0.28); p =0.565
EsoWall in PTV D0.1cc (Gy)-Change Percent (%)

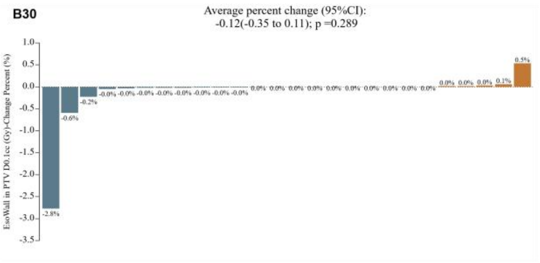
B30
Average percent change (95%CI):
-0.12(-0.35 to 0.11); p =0.289
EsoWall in PTV D0.1cc (Gy)-Change Percent (%)

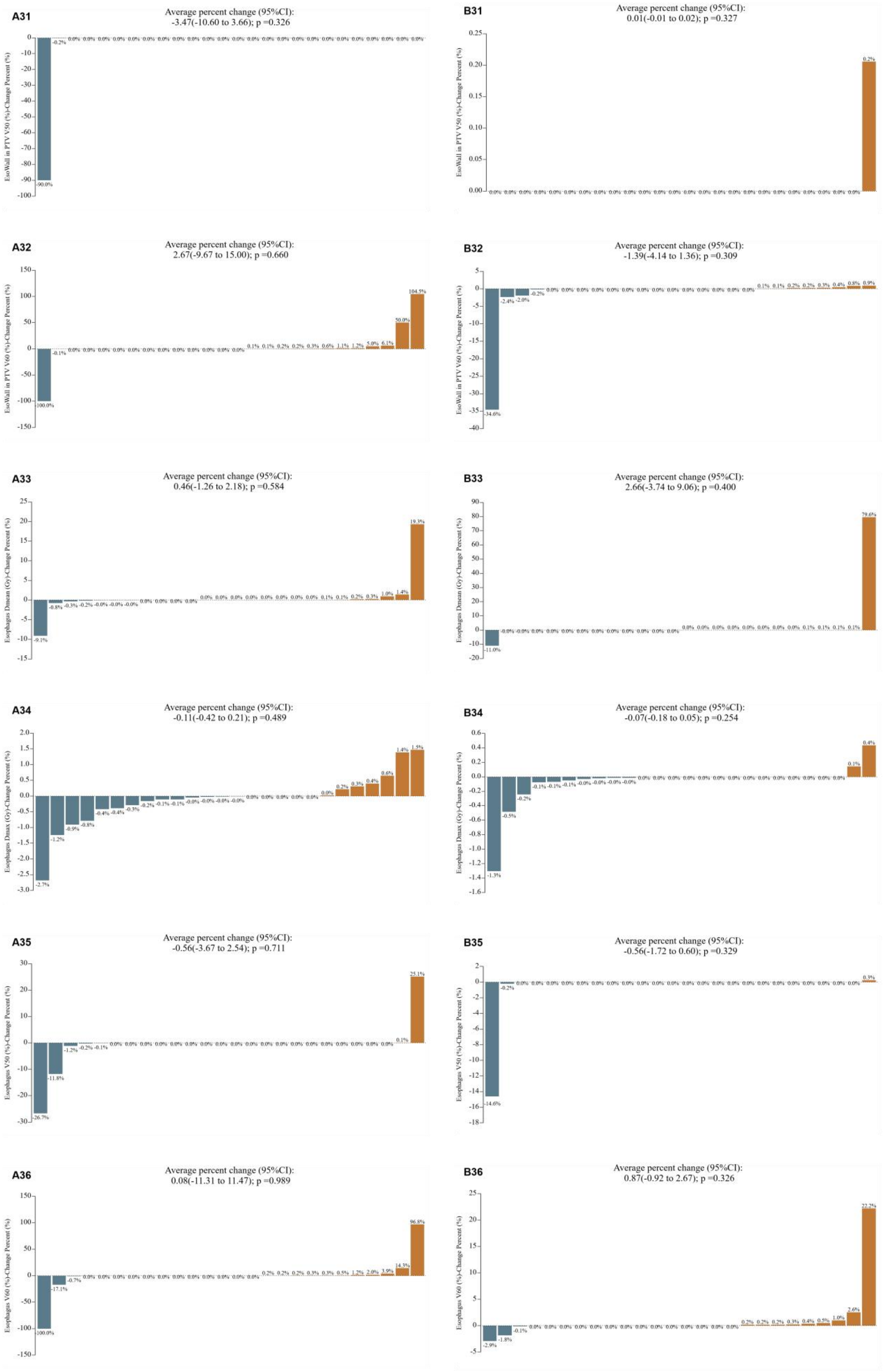

Supplementary Figure S3. Waterfall plots illustrating the percentage dose changes (Δ%) between native and override plans for photon (A panels) and proton (B panels) therapies. Bars are color-coded

according to the direction of change: gray blue indicates dose decreases (Δ < 0), and orange indicates dose increases (Δ > 0). The same color scheme was applied for both photon and proton groups for consistency. Each bar represents one patient. Mean change (95% confidence interval) and p-values were derived from paired t-tests.

Supplementary Table S4. Linear mixed-effects model results evaluating the interaction between radiotherapy technique and plan type on dose perturbation metrics

Structure / Metric	Effect	Estimate (β)	95% CI	p
PTV D95 (%)	Modality × ClipState	-0.03	(-1.83, 1.76)	0.973
PTV Dmax (Gy)	Modality × ClipState	0.06	(-0.95, 1.07)	0.902
PTV with Clip D95 (Gy)	Modality × ClipState	-0.30	(-0.89, 0.29)	0.327
PTV with Clip Dmax (Gy)	Modality × ClipState	-0.25	(-1.37, 0.86)	0.658
GTV D95 (%)	Modality × ClipState	-0.13	(-0.93, 0.67)	0.749
GTV Dmax (Gy)	Modality × ClipState	0.03	(-1.10, 1.15)	0.963
GTV with Clip D95 (Gy)	Modality × ClipState	-0.36	(-0.93, 0.20)	0.215
GTV with Clip Dmax (Gy)	Modality × ClipState	0.06	(-0.99, 1.10)	0.914
Clip Dmean (Gy)	Modality × ClipState	-0.8	(-1.48, -0.12)	0.024
Clip Dmax (Gy)	Modality × ClipState	-0.22	(-1.19, 0.74)	0.649
Clip D0.1cc (Gy)	Modality × ClipState	-0.57	(-1.35, 0.21)	0.154
Clip 5mm Dmean (Gy)	Modality × ClipState	-0.08	(-0.60, 0.45)	0.773
Clip 5mm Dmax (Gy)	Modality × ClipState	-0.03	(-1.10, 1.03)	0.954
Clip 5mm D0.1cc (Gy)	Modality × ClipState	0.17	(-0.81, 1.15)	0.735
Clip 5mm in EsoWall Dmean (Gy)	Modality × ClipState	-0.14	(-0.73, 0.46)	0.652
Clip 5mm in EsoWall Dmax (Gy)	Modality × ClipState	0.04	(-0.95, 1.03)	0.934
Clip 5mm in EsoWall D0.1cc (Gy)	Modality × ClipState	0.15	(-0.77, 1.07)	0.752
Clip 3mm Dmean (Gy)	Modality × ClipState	-0.15	(-0.70, 0.39)	0.583
Clip 3mm Dmax (Gy)	Modality × ClipState	-0.05	(-1.06, 0.97)	0.927
Clip 3mm D0.1cc (Gy)	Modality × ClipState	0.00	(-0.84, 0.85)	0.998
Clip 3mm in EsoWall Dmean (Gy)	Modality × ClipState	-0.16	(-0.78, 0.45)	0.602
Clip 3mm in EsoWall Dmax (Gy)	Modality × ClipState	0.04	(-0.96, 1.04)	0.938
Clip 3mm in EsoWall D0.1cc (Gy)	Modality × ClipState	-0.13	(-0.91, 0.66)	0.754
EsoWall Dmean (Gy)	Modality × ClipState	-0.17	(-1.11, 0.76)	0.716
EsoWall Dmax (Gy)	Modality × ClipState	0.06	(-1.12, 1.24)	0.924
EsoWall V50 (%)	Modality × ClipState	-0.02	(-1.23, 1.19)	0.975
EsoWall V60 (%)	Modality × ClipState	-0.27	(-5.20, 4.66)	0.915
EsoWall in PTV Dmean (Gy)	Modality × ClipState	-0.20	(-0.81, 0.41)	0.517
EsoWall in PTV Dmax (Gy)	Modality × ClipState	0.07	(-1.03, 1.18)	0.897
EsoWall in PTV D0.1cc (Gy)	Modality × ClipState	-0.11	(-1.12, 0.90)	0.83
EsoWall in PTV V50 (%)	Modality × ClipState	0.08	(-0.20, 0.37)	0.556
EsoWall in PTV V60 (%)	Modality × ClipState	-0.63	(-9.97, 8.70)	0.894
Esophagus Dmean (Gy)	Modality × ClipState	1.02	(-1.37, 3.40)	0.407
Esophagus Dmax (Gy)	Modality × ClipState	0.03	(-1.13, 1.18)	0.966
Esophagus V50 (%)	Modality × ClipState	-0.21	(-1.53, 1.12)	0.76
Esophagus V60 (%)	Modality × ClipState	0.06	(-4.70, 4.81)	0.981

β represents the estimated coefficient from linear mixed-effects models assessing the interaction between radiotherapy technique and plan type.

Abbreviations: Gy, gray; D95, dose to 95% of the volume; Dmean, mean dose; Dmax, maximum dose; D0.1cc, minimum dose delivered to the hottest 0.1 cc; EsoWall, esophageal wall.

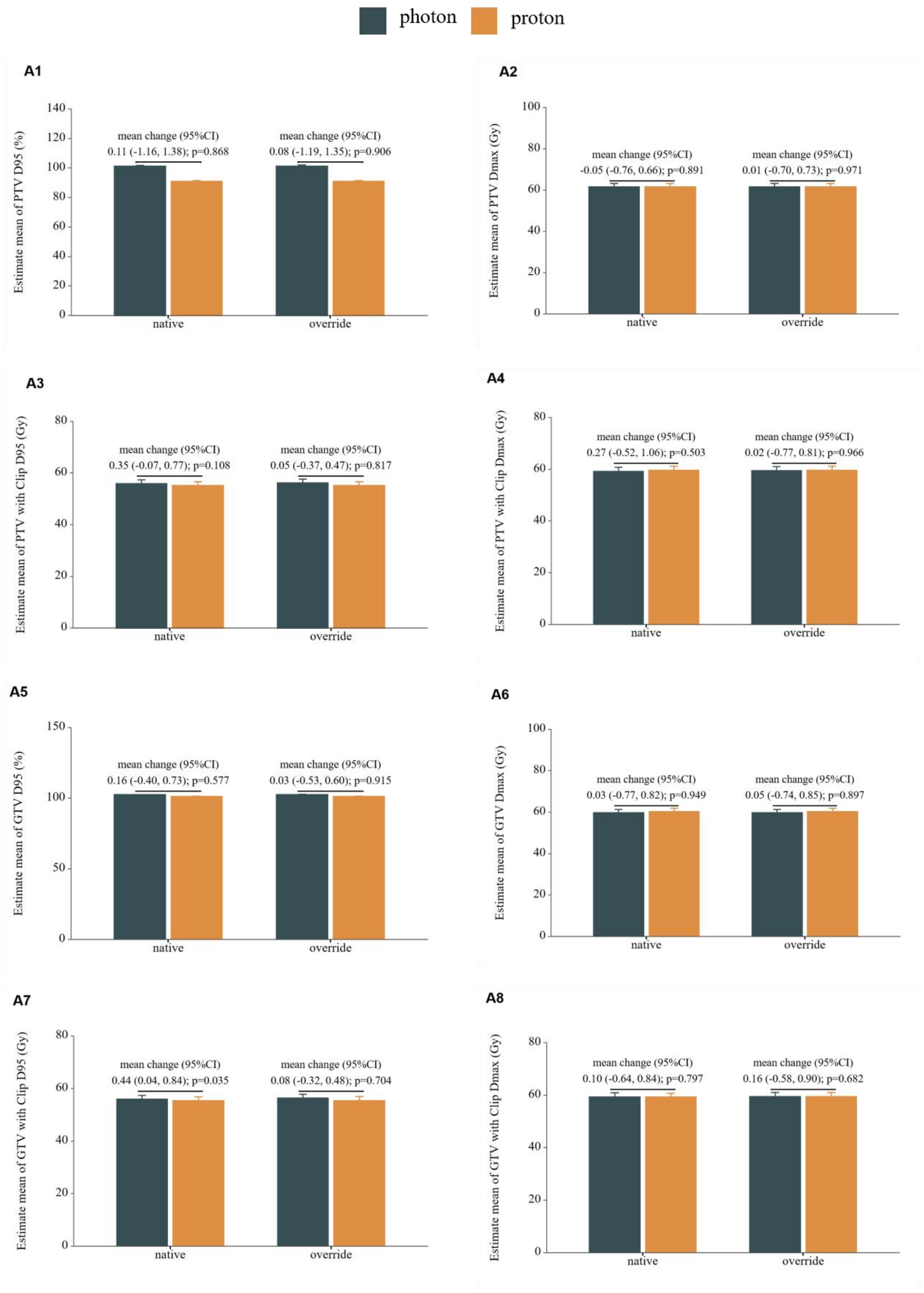
photon
proton
A1
Estimate mean of PTV D95 (%)
mean change (95%CI)
0.11 (-1.16, 1.38); p=0.868
mean change (95%CI)
0.08 (-1.19, 1.35); p=0.906
0
20
40
60
80
100
120
140
native
override
A2
Estimate mean of PTV Dmax (Gy)
mean change (95%CI)
-0.05 (-0.76, 0.66); p=0.891
mean change (95%CI)
0.01 (-0.70, 0.73); p=0.971
0
20
40
60
80
100
native
override
A3
Estimate mean of PTV with Clip D95 (Gy)
mean change (95%CI)
0.35 (-0.07, 0.77); p=0.108
mean change (95%CI)
0.05 (-0.37, 0.47); p=0.817
0
20
40
60
80
native
override
A4
Estimate mean of PTV with Clip Dmax (Gy)
mean change (95%CI)
0.27 (-0.52, 1.06); p=0.503
mean change (95%CI)
0.02 (-0.77, 0.81); p=0.966
0
20
40
60
80
native
override
A5
Estimate mean of GTV D95 (%)
mean change (95%CI)
0.16 (-0.40, 0.73); p=0.577
mean change (95%CI)
0.03 (-0.53, 0.60); p=0.915
0
50
100
150
native
override
A6
Estimate mean of GTV Dmax (Gy)
mean change (95%CI)
0.03 (-0.77, 0.82); p=0.949
mean change (95%CI)
0.05 (-0.74, 0.85); p=0.897
0
20
40
60
80
100
native
override
A7
Estimate mean of GTV with Clip D95 (Gy)
mean change (95%CI)
0.44 (0.04, 0.84); p=0.035
mean change (95%CI)
0.08 (-0.32, 0.48); p=0.704
0
20
40
60
80
native
override
A8
Estimate mean of GTV with Clip Dmax (Gy)
mean change (95%CI)
0.10 (-0.64, 0.84); p=0.797
mean change (95%CI)
0.16 (-0.58, 0.90); p=0.682
0
20
40
60
80
native
override

A9

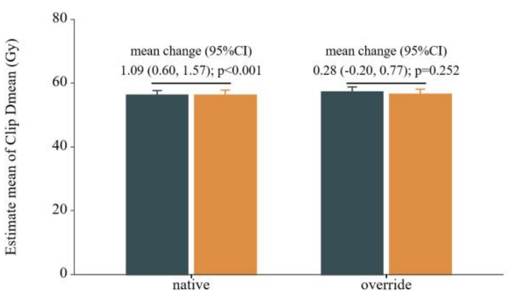

A10

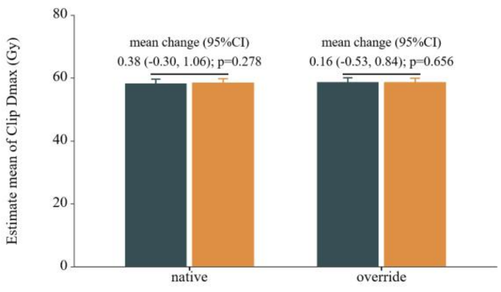

A11

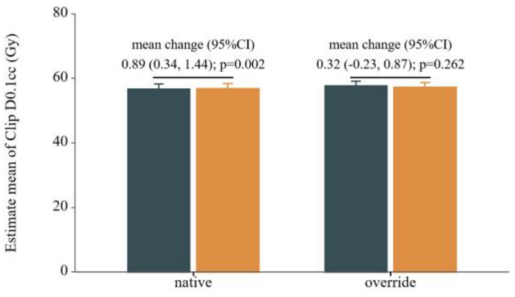

A12

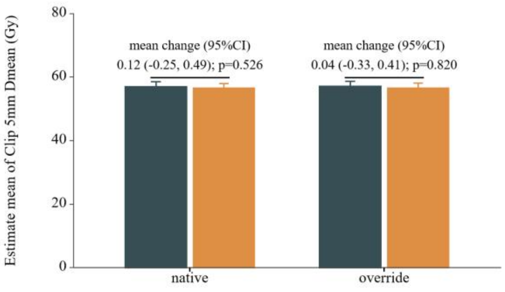

A13

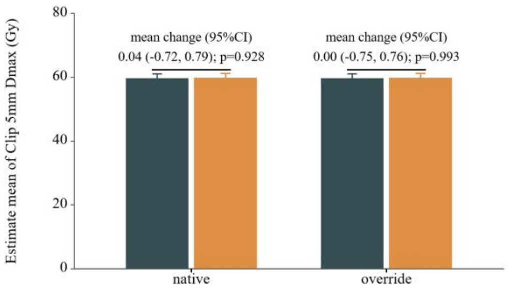

A14

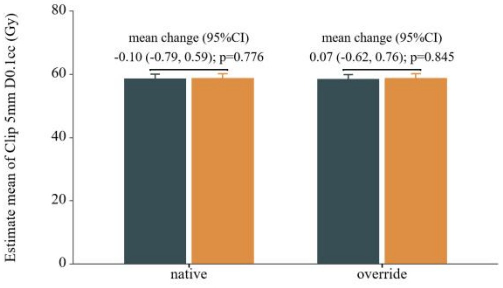

A15

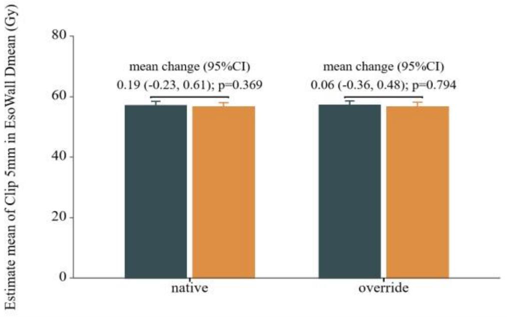

A16

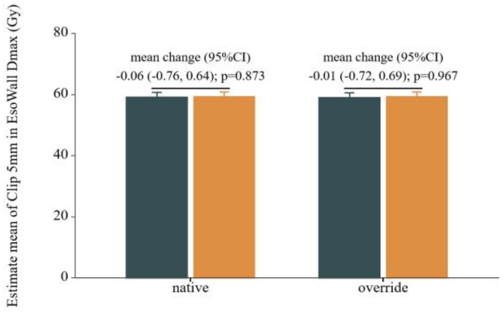

A17

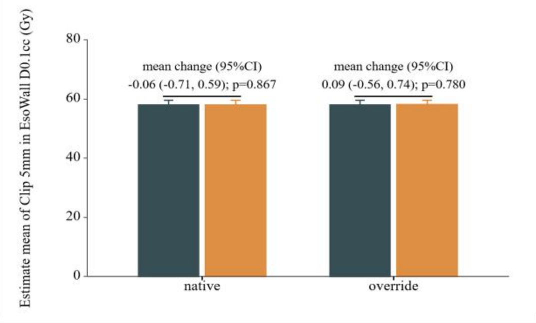

A18

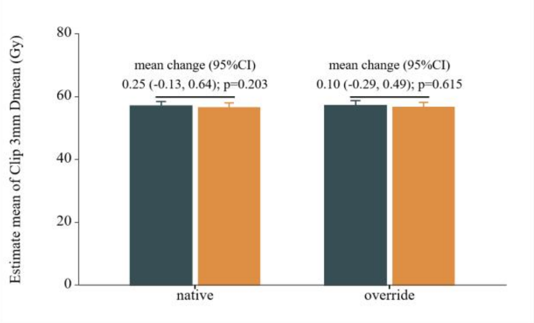

A19

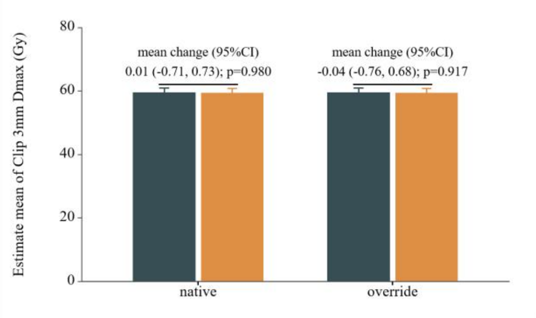

A20

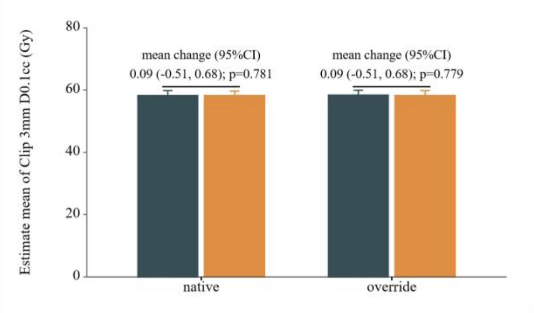

A21

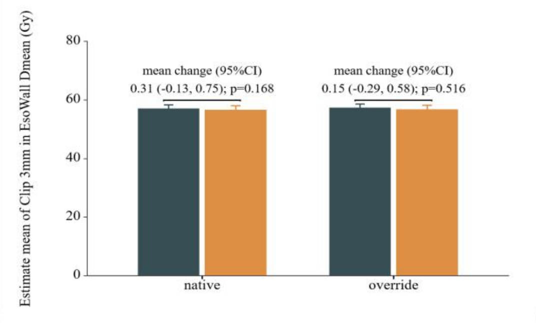

A22

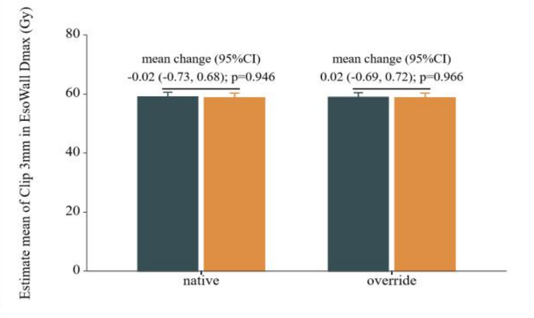

A23

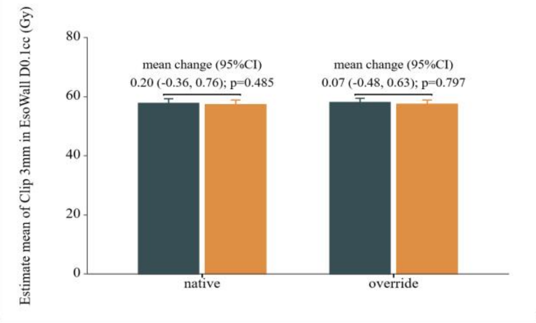

A24

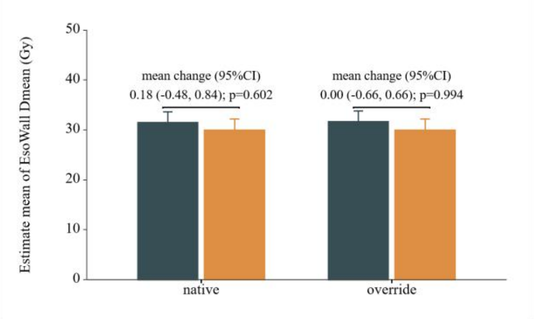

A25

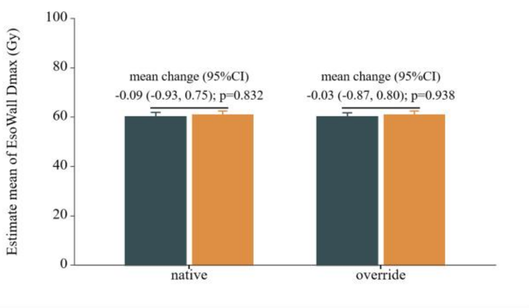

A26

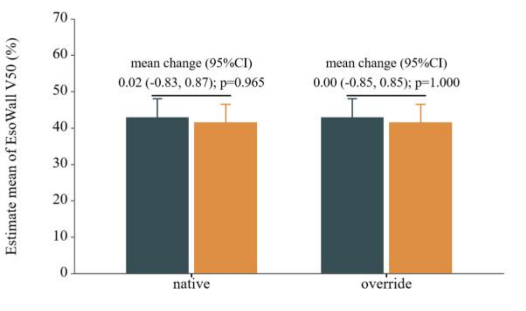

A27

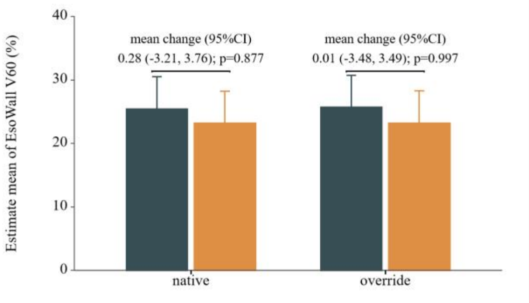

A28

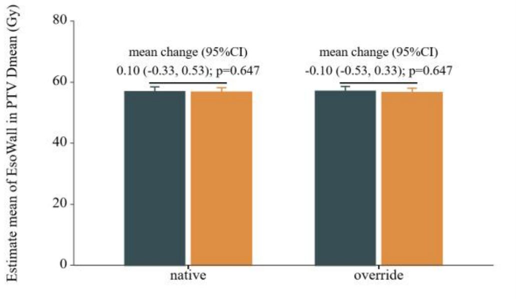

A29

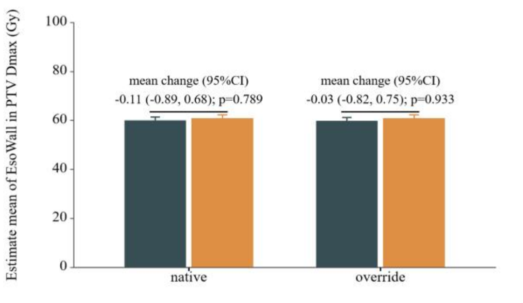

A30

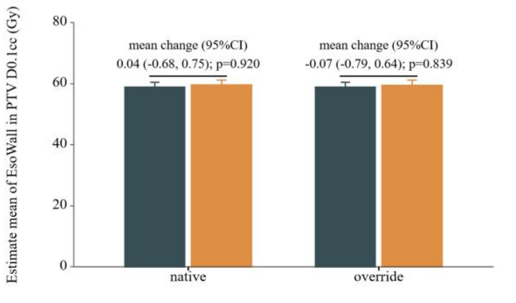

A31

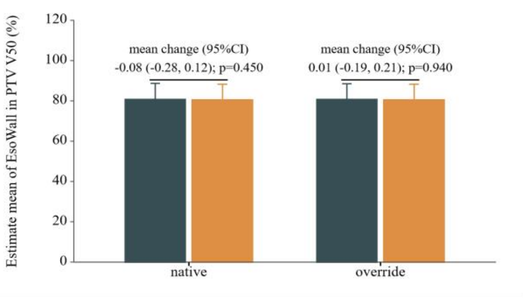

A32

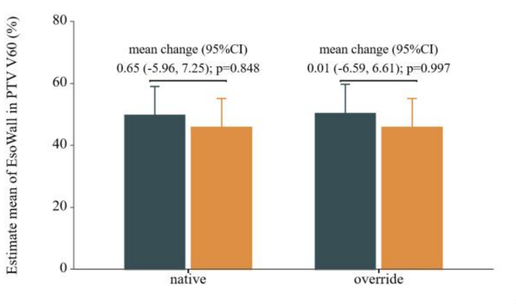

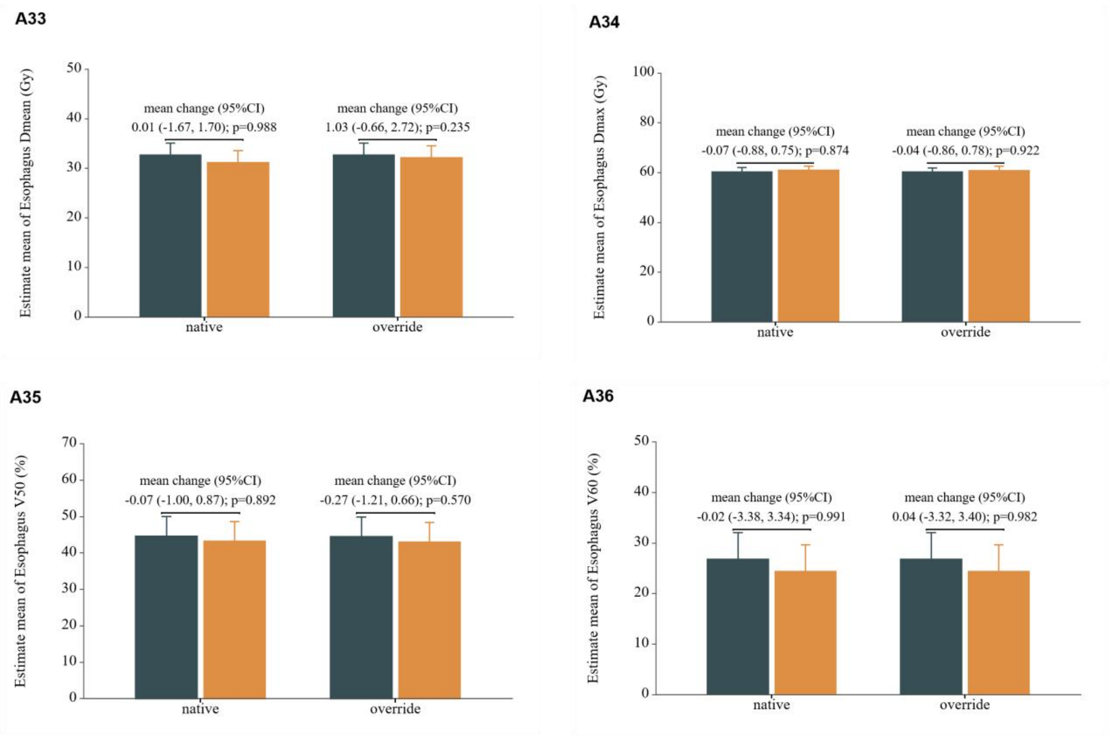

Supplementary Figure S4. Estimated mean doses (Gy) from the linear mixed-effects model for photon and proton therapies, stratified by plan type (native vs override). Bars are color-coded by technique (darker gray for photon, orange for proton). Error bars represent 95% confidence intervals. Mean change (95% CI) and p-values were derived from the mixed-effects model.
